# Gene Co-Expression Modules as Clinically Relevant Hallmarks of Breast Cancer Diversity

**DOI:** 10.1371/journal.pone.0088309

**Published:** 2014-02-07

**Authors:** Denise M. Wolf, Marc E. Lenburg, Christina Yau, Aaron Boudreau, Laura J. van ‘t Veer

**Affiliations:** 1 Department of Laboratory Medicine, University of California San Francisco, San Francisco, California, United States of America; 2 Department of Medicine, Section of Computational Biomedicine, Boston University School of Medicine, Boston, Massachusetts, United States of America; 3 Department of Surgery, University of California San Francisco, San Francisco, California, United States of America; 4 Buck Institute for Research on Aging, Novato, California, United States of America; Institut de recherches cliniques de Montréal (IRCM), Canada

## Abstract

Co-expression modules are groups of genes with highly correlated expression patterns. In cancer, differences in module activity potentially represent the heterogeneity of phenotypes important in carcinogenesis, progression, or treatment response. To find gene expression modules active in breast cancer subpopulations, we assembled 72 breast cancer-related gene expression datasets containing ∼5,700 samples altogether. Per dataset, we identified genes with bimodal expression and used mixture-model clustering to ultimately define 11 modules of genes that are consistently co-regulated across multiple datasets. Functionally, these modules reflected estrogen signaling, development/differentiation, immune signaling, histone modification, ERBB2 signaling, the extracellular matrix (ECM) and stroma, and cell proliferation. The Tcell/Bcell immune modules appeared tumor-extrinsic, with coherent expression in tumors but not cell lines; whereas most other modules, interferon and ECM included, appeared intrinsic. Only four of the eleven modules were represented in the PAM50 intrinsic subtype classifier and other well-established prognostic signatures; although the immune modules were highly correlated to previously published immune signatures. As expected, the proliferation module was highly associated with decreased recurrence-free survival (RFS). Interestingly, the immune modules appeared associated with RFS even after adjustment for receptor subtype and proliferation; and in a multivariate analysis, the combination of Tcell/Bcell immune module down-regulation and proliferation module upregulation strongly associated with decreased RFS. Immune modules are unusual in that their upregulation is associated with a good prognosis without chemotherapy *and* a good response to chemotherapy, suggesting the paradox of high immune patients who respond to chemotherapy but would do well without it. Other findings concern the ECM/stromal modules, which despite common themes were associated with different sites of metastasis, possibly relating to the “seed and soil” hypothesis of cancer dissemination. Overall, co-expression modules provide a high-level functional view of breast cancer that complements the “cancer hallmarks” and may form the basis for improved predictors and treatments.

## Introduction

The dream of personalized oncology has every woman diagnosed with breast cancer matched with the treatment most likely to save her life, without either under- or over-treatment. Impeding the attainment of this dream is the complex, heterogeneous nature of breast cancer, with wildly variable histology, morphology, hormone receptor and HER2 expression, progression tempo, risk of recurrence, and patterns of dissemination during metastatic recurrence, much of which affects the need for and response to systemic therapies. Differences in breast cancer biology and prognosis are demonstrably reflected in underlying differences in gene expression; indeed, variability in transcriptomic profiles were first observed and summarized into five well-defined intrinsic molecular tumor subtypes in Perou’s landmark study in 2000 [1], [2], a classification largely recapitulated in the recent much larger TCGA study incorporating protein expression, DNA methylation, copy number aberrations, and microRNA expression [3]. Other studies have produced different but related molecular definitions of breast cancer heterogeneity, expanding the catalog of breast cancer to perhaps ten molecular subtypes [4].

This study is an effort to further functionally characterize breast cancer heterogeneity through the concept of modules; we hypothesize that such modular decomposition could yield clinically actionable components useful in achieving the goals of personalized oncology. Many definitions for biological modules have been proposed over the years [5], [6]; what unifies these definitions is that they attempt to simplify complex systems with large webs of interacting components into a smaller set of functionally integrated themes. The canonical ‘hallmarks of cancer’, while primarily describing fundamental processes of carcinogenesis, can also be viewed as an informal attempt to impose or extract a modular structure on the complexity of cancer dynamics [7], [8]. According to this paradigm, the hallmarks of cancer include sustaining proliferative signaling, evading growth suppressors, resisting cell death, enabling replicative immortality, inducing angiogenesis, and activating invasion and metastasis (the original six). To these six, a recent extension has added the reprogramming of energy metabolism and evading immune destruction, with emphasis placed on the interplay between malignant and hijacked ‘normal’ cells in the tumor microenvironment [8].

The growing number of breast cancer related genome-wide gene-expression profiling datasets provides an opportunity to perform a comprehensive search for common patterns of gene co-expression using a formal, computable approach to distinguish different gene programs in breast cancer. Such co-expression modules can be viewed as an empirically derived catalog of coherent gene groups that might act together, and may have been selected for, as a unit to perform a function important to the cancer. Thus, the activity of modules within a tumor may be useful in understanding how that cancer developed, its likelihood of distant recurrence without systemic treatment, and potential vulnerabilities that may be targeted by therapeutics [9], [10], [11], [12], [13], [14], [15], [16], [17]. A prior study comparing the genomic and transcriptomic profiles of normal and malignant breast identified 16 modules, one enriched for proliferation and two for immune response [10]. Other studies have interrogated transcriptomic profiles for associations between co-expressed gene clusters and grade [12], aberrant chromosomal regions [13], and tumorigenesis [15], among others. Though not explicitly termed ‘modules’, many other breast cancer gene expression studies implicitly rely on and address gene expression modularity by identifying cohesive gene expression clusters observable in unsupervised hierarchical clustering, followed by gene set enrichment to assign pathway-activation patterns that may be associated with a phenotype [1], [2].

In this study, we compiled a large repository of publicly available transcriptomic data totaling 5,684 samples, obtained from breast cancer patients and breast cancer cell lines, to identify breast cancer co-expression modules. Cell lines capture much of the heterogeneity observed in human tumors [18], and datasets representing genetically and chemically perturbed cell lines may yield modules important to treatment response to targeted therapeutics. We identified a total of 11 conserved modules, many of which are enriched for genes involved in at least one of the canonical ‘hallmarks of cancer’ [7], [8]. We then correlated the expression levels of these modules to recurrence-free survival, site-specific RFS and metastasis, chemotherapy response, and multiple signatures [19], [20], [21], [22]. We observed that whereas some of the modules were similar to signatures previously linked with breast cancer heterogeneity and therapy response, others appeared unique. Activity of several of the unique modules was associated with patient outcome, site of metastasis, or chemo-sensitivity. We also assessed the level of heterogeneous expression and co-regulation of the modules in breast cancer cell lines (BCCL), providing insight into which in vivo genetic programs are most and least represented by the BCCL forming the underpinnings of most in vitro breast cancer studies. Viewed as a whole, our results suggest that meta-analysis of breast-cancer related gene expression data can be used to identify robust and potentially novel patterns of gene co-expression that may highlight interesting cancer biology and be useful for guiding treatment strategies.

## Results

### Breast Cancers are Distinguished via Common Transcriptional Modules

We obtained 72 breast cancer gene expression datasets from the Gene Expression Omnibus (GEO) and several other public sources (see File S1) consisting of 5684 samples profiled on Affymetrix U133-type arrays. Overall, this data compendium represents normal breast tissue, breast tumors of every subtype, laser micro-dissected (LMD) breast tumor epithelium and stroma, pre-treatment tumors, post-treatment tumors, a few metastases, and a variety of breast cancer cell lines, including genetically perturbed derivatives, grown in serum or subjected to chemotherapies or other compounds. We identified the subset of genes with a strong bimodal gene expression pattern across the samples in each dataset, motivated by the hypothesis that a pattern of “on or off” gene expression was much more likely due to biological control rather than technical factors. Using unsupervised clustering of genes with bimodal expression within each dataset, we identified in total 683 clusters of co-regulated genes across the compendium, as described in the Methods section.

**Figure 1:**
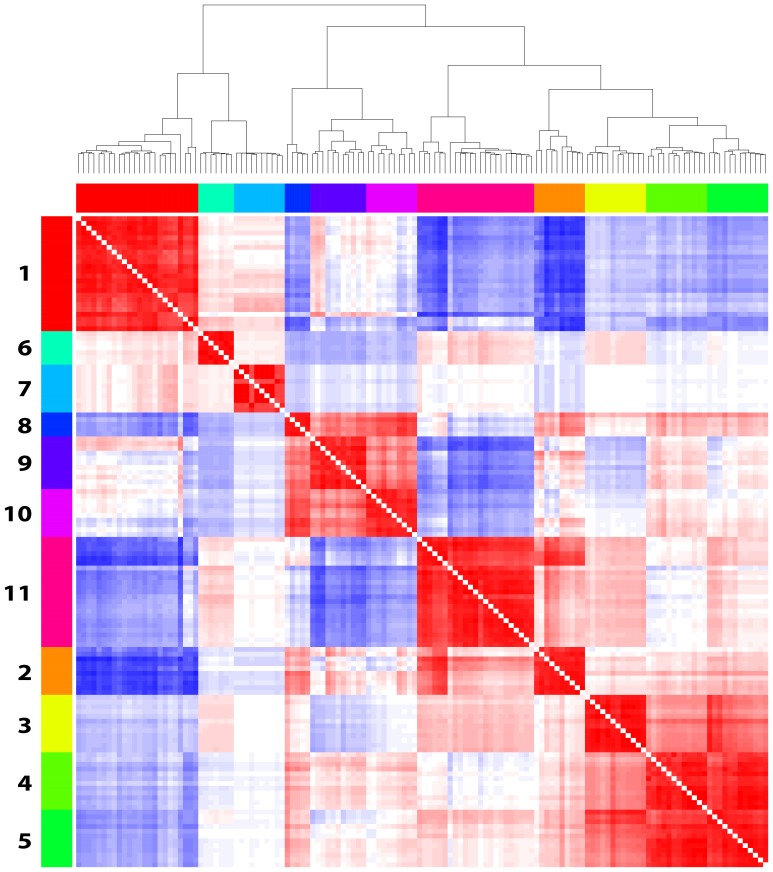
Clustered heat map used to define breast cancer co-expression modules. Cross-correlation heat map of the 136 robust signatures derived from 72 datasets cluster into 11 coexpression modules.

We scored all samples in the compendium of datasets for the activity of each of the 683 clusters using principal component analysis, and identified 136 clusters that produced highly correlated scores with at least six other clusters, which we selected to represent recurrent patterns of similarly coordinated gene expression. When we aggregated these highly correlated clusters, 11 co-expression modules consisting of 5–23 clusters were observed (Figure 1). We validated the clustering in Figure 1 using SigClust [23] (with 1000 simulations, the “hard thresholding” method reported by Liu et al. for estimating the eigenvalues of the covariance matrix [23], and p-values determined empirically from the simulated null distribution) to determine if each of the modules was distinct from the other modules. Using this method, we obtained p-values <0.001 for all pairwise comparisons except for Module 8 compared to Module 10 (p = 0.478). Using the more recently described “soft thresholding” method for estimating the eigenvalues of the covariance matrix used by SigClust [24], all of the pairwise module comparisons were significant (p<0.001). Because of the soft-thresholding results and the biological differences we observed between Module 8 and Module 10 (described below) we chose to maintain the distinction between Modules 8 and 10.

These cluster filtering and aggregation steps were based on the hypothesis that important breast-cancer related biological differences between samples were likely to be present in multiple datasets. Selecting only the genes that appeared in more than 33% of the clusters comprising a given module, the 11 co-expression modules were distilled to contain 4 to 247 genes that exhibit a strong on-off expression between breast cancer samples and are consistently co-expressed across multiple datasets (see File S1 for gene lists). We hypothesize that these modules represent functionally coherent biological differences between breast tumors that may highlight important biology and have clinical applications.

### Modules are Enriched for Specific Functions and Pathways

Functional/pathway enrichment analysis of the 11 co-expression modules in breast cancer using DAVID [25], g:Profiler [26], and a manual literature search suggests that as expected, estrogen signaling (Module 1), cell proliferation (Module 11), and ERBB2 signaling (Module 7) are represented; in addition, we found modules associated with immune signaling (Modules 3–5), development/differentiation (Module 2), histone modification (Module 6), and the ECM (Module 10), as well as two stromal wound repair/angiogenesis modules combining microenvironment, developmental and immune genes (Modules 8 and 9).

The estrogen signaling module 1 (1-ER) contains 135 genes, among them ESR1 and a plethora of genes known to be regulated by estrogen. Module 1 also contains androgen receptor (AR) and ERBB4, a component of the Her2 signaling cascade associated with endocrine resistance [27] and sensitivity to the MTOR inhibitor everolimus [28]. Module 7 (7-ERBB2), the ERBB2 signaling module, contains only 4 genes and is essentially a minimal ERBB2 amplicon in Her2+ breast cancer. Proliferation module 11 (11-Proliferation) contains 120 genes functionally enriched for cell cycle mitosis, checkpoints, meiosis, and DNA replication. Immune modules 4 and 5 (4-Immune, 5-Immune) contain 82 and 80 genes, respectively. Both are highly enriched for immune response functions and pathways, with 4-Immune leaning toward T cell and B cell activation, and 5-Immune more enriched for chemokine signaling, defense, and inflammatory responses. Immune module 5 (5-Immune) has a partial overlap (35%) with 4-Immune, but differs from 4-Immune in that it includes many more chemokines and interleukins capable of inducing TNFalpha from immune and epithelial cells. The third immune module (3-Immune IFN) contains 25 genes, most involved in interferon-mediated signaling. Module 6 (6-Histone) is small, with just 12 genes all belonging to histone families involved in nucleosome assembly and organization, chromatin assembly, and telomere maintenance. Module 2 (2-Dev/basal; 247 genes) contains a mix of mostly basal cytokeratins, cell:cell adhesion genes, integrins matrix metallopeptidases, and other cell differentiation genes, yielding a functional enrichment for developmental processes. Module 10 (10-ECM) represents extracelluar matrix (ECM) genes and processes. Modules 8 and 9 are associated with stromal wound repair/angiogenesis, with Module 8 dominated by genes involved in hemostasis and blood vessel morphogenesis and wound response, and Module 9 (9-ECM/Dev/Immune) a mix of ECM, muscle/myeloid development, and inflammatory response genes.

**Figure 2:**
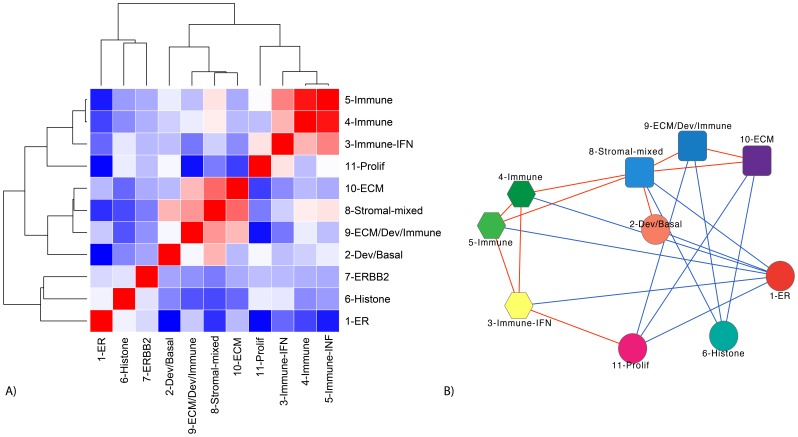
Module correlation patterns. A) A clustered heatmap of Pearson correlation coefficients over all module pairs (using Pearson distance, and average linkage). Dark red denotes high correlation (r → 1), dark blue high anti-correlation (r→ −1), and white a lack of correlation (r ≅ 0). B) This network representation of (A) illustrates the correlation and anti-correlation topology of module expression; red links denote module pairs with Pearson correlation coefficients r >0.25, whereas blue links denote module pairs with r<−0.25. These figures represent the covariance of ∼3700 samples from 24 datasets listed in File S1.

Functional enrichments and representative genes for each of the modules are summarized in Table 1, and a complete list of module genes can be found in File S1. Examples of the coordinate differential expression of module genes in different breast cancer datasets are shown in Figure S1 in File S2, and covariance patterns among the modules are shown in Figure 2. Consistent with other publications, a low level of estrogen signaling (1-ER) is associated with high proliferation (11-Prolif) and basal (2-Dev/Basal) gene expression [1], [2], and high immune signaling (3∶5-Immune) [29], the latter of which is associated with improved outcomes [30], [31] (Figure 2B).

**Table 1:** Functional enrichment of modules.

Module	Size(# genes)	Pathway/functional enrichment (%FDR)	Representative genes
**1-ER**	135	Estrogen signaling, cell-cell signaling (0.045), hormone estrogen/stimulus (2.24)	ESR1, PGR, FOXA1, GATA3, TFF1, TFF3; TFAP2B; SLCA1; AGTR1, MAPT, MUC1, AR, ERBB4
**2-Dev/basal**	247	Ectoderm development (9.15E-04), epidermis development(0.055), cell adhesion (0.063), vitamin metabolic process (0.63)	KRT5, 7, 14; DSC2, DSC3; ITGB6, ITGB8; ITGB6,8; COL2A, DKK1, ELF5, EN1, FOXC1, GATA6, GJB5
**3-Immune-IFN**	28	Response to type 1 interferon, cytokine mediated signaling,immune response (1.93E-06), response to virus (3.37E-06),RIG-I like receptor signaling (.015),DNA replication and repair (0.2)	STAT1; IFI27, IFI44, IFI44L, IFI6, IFIH1, IFIT1, IFIT2, IFIT3
**4-Immune**	82	Immune response (1.15E-18), lymphocyte activation (3.3E-13), leukocyte activation (7.31E-12), positive regulation of immune system (1.76E-09), T cell activation (2.7E-05)	CD2, CD8A, CD7, CD3G; GZMA,B,K; TNFRSF17, CD27; IL21R, IL2R; CCR2, CCR7, CCL19
**5-Immune**	80	Immune response (4E-24), defense response (1.2E-14),inflammatory response (1.01E-07), chemokine signaling(9.23E-06), cytokine-cytokine receptor interaction (4.33E-06)	CCL13, CCL18, CCL19, CCL5, CCL8, CXCL10, CXCL12, CXCL13, CXCLL9, CXCLR6, IRF1, IL32
**6-Histone**	12	Nucleosome assembly (1.27E-15), chromatin assembly (1.71E-15), protein-DNA complex assemply (2.48E-15), nucleosomeorganization (2.98E-15), telomere maintenance (1.7E-10), lupus	HIST1H1C, HIST1H2AE, HIST1H3D, HIST1H4H, HIST2H2BE
**7-ERBB2**	4	ERBB2 amplicon. EGFR signaling pathway, EGFR activity,ErbB3 class receptor binding, oncogenomic recombinationhotspot (0.001)	ERBB2, GRB7, STARD3, PGAP3
**8-Stromal-mixed**	82	Regulation of cell proliferation (0.001), regulation of signal transduction (0.019), Hemostasis (0.02), blood vessel morphogenesis/development (0.11), response to wounding (0.36)	FBN2, PLAT, SERPINE1, L1CAM; TGFB2, VIM, LYN, BONF, CAV1, CAV2, DKK1, FOXF2, IGFBP6
**9-ECM/Dev/** **Immune**	110	Extracellular region/part/space (6.41E-10), response to hormone stimulus (3.5E-05), extracellular matrix (3.89E-05), regulation of inflammatory response (0.001), muscle organ development(0.0027)	RBP4, TF, CXCL2, TIMP4, ADIPOQ, CHRDL1, EDNRB, FABP4, FIGF, GPC3, IL6; HOXA10, HOXA5, HOXA7; WIF1, GHR, IGF1, PPARG
**10-ECM**	58	Proteinaceous ECM (2.7E-18), ECM (1.3E-17), collagen (6.1E-10),ECM-receptor interaction (2.2E-09), cell adhesion (4.7E-10)),signaling by PDGF (2.3E-07),focal adhesion (2.1E-07)	ASPN, COL1A1, COL3A1, COL5A1, COL6A1-3; FBN1, FN1, LOX, LUM, NID1;FAP; CDH13, GPR124, PDGFRA
**11-Prolif**	120	Cell cycle mitotic (5.87E-30), cell cycle (1E-16), cell cyclecheckpoints (1.94E-09), oocyte meiosis (4.84E-06),KISc (1.04E-05), p53 signaling (0.0091), DNA replication (0.12)	CCNB1, CCNB2, CCNE2, MKI67, AURKA-B, E2F8, CENPA, KIF11, TOP2A, HJURP, RAD21, RAD51AP

This table summarizes the pathway and functional themes of the genes in each module, obtained from applying DAVID and g:Profiler algorithms. FDR = false discovery rate (reported for DAVID results).

### Some Modules Correlate to Clinical Biomarkers of Breast Cancer whereas Immune, Histone, and ECM Modules Appear Novel

To evaluate whether the modules identified in this study are represented in current intrinsic subtype classifiers (PAM50 [32]) and prognostic signatures clinically in use to differentiate breast cancers (70-gene prognosis signature [33], and 21-gene recurrence score [34]), we first quantified the overlap between the 958 genes comprising our 11 co-expression modules and the genes within these three signatures. We found that of the 48 evaluable genes in the PAM50 intrinsic subtype classifier, 30 (62.5%) overlap with genes in Modules 1-ER, 11-Prolif, 7-ERBB2 or 2-Dev/Basal. Similarly, 10 of the 16 (62.5%) and 12 of the 70 (17%) evaluable genes in the 21-gene recurrence score and the 70-gene prognosis signature, respectively, are distributed among the estrogen signaling (1-ER), proliferation (11-Prolif), ERBB2 (7-ERBB2) and/or developmental (2-Dev/Basal) modules. Genes from 7 of the 11 breast cancer co-expression modules (immune modules 3–5, histone module 6-Histone, the mixed modules 8-mixed and 9-ECM/Dev/Immune, and the ECM module 10-ECM) are not represented in these three signatures (Table 2).

**Table 2:** Overlap between module genes and established signatures.

Module	PAM50 (Intrinsic subtypes)	70-gene prognosis signature (MammaPrint™)	21-gene recurrence score (Oncotype DX)
**1-ER**	**ESR1**, FOXA1, MLPH, **PGR**, NAT1, SLC39A6	**SCUBE2**	**ESR1, PGR, SCUBE2**
**2-Dev/Basal**	FOXC1, MIA, SFRP1, KRT14, KRT5, CDH3	NMU, HRASLS, TSPYL5	
**7-ERBB2**	**ERBB2, GRB7**		**ERBB2, GRB7**
**11-Prolif**	CEP55, **MELK**, CDC20, ACTR38, **CCNB1**, CDC6, RRM2, KIF2C, **MKI67**, UBE2C, PTTG1, EXO1, **MYBL2**, **BIRC5**	CENPA, NMU, ECT2, NUSAP1, GPR126, RFC4, PRC1, **MELK**	**MKI67**, **MYBL2**, **BIRC5**, **CCNB1**, AURKA
**Total**	30/48 (62.5%)	12/54 (22%)	10/16 (62.5%)

Modules 1-ER, 11-Prolif, 7-ERBB2 and 2-Dev/Basal share genes in common with the PAM50 gene set used to evaluate intrinsic subtype, the NKI70 prognostic classifer used in MammaPrint, or the 21-gene prognostic signature used in OncotypeDX. Immune modules 3–5, histone module 6-Hist, the stromal modules 8 and 9-ECM/Dev/Immune, and the ECM module 10-ECM have no genes in common with these signatures. Listed genes are present in both the specified module and the labeled signature. Genes in bold face are present in the module and multiple signatures.

**Figure 3:**
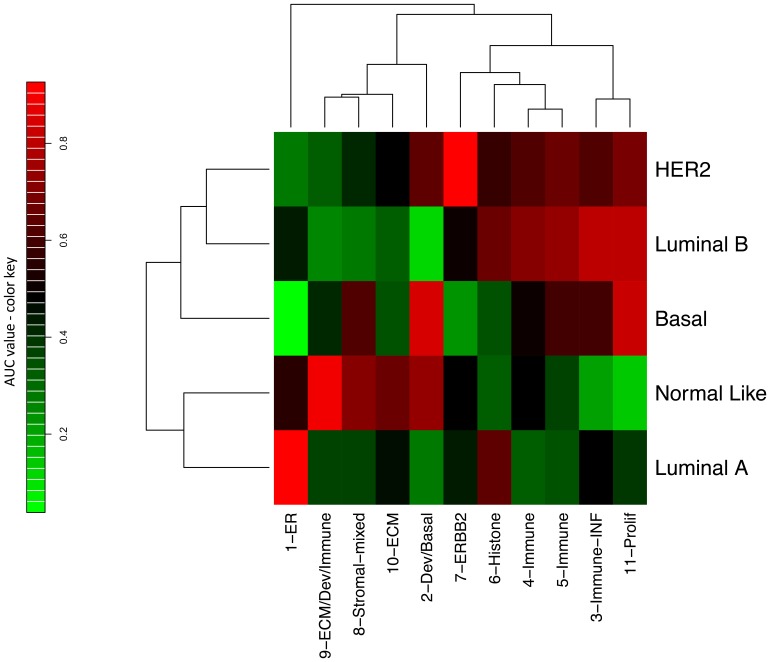
Modules vs. intrinsic subtype heatmap. This heatmap shows hierarchically clustered AUC scores summarizing how well each intrinsic subtype can be predicted by each coexpression module score. Red denotes high positive predictive value (AUC → 1), green high negative predictive value (AUC → 0), and black a non-informative relationship (AUC≈0.5). This figure represents GSE1456, with AUC’s clustered using Euclidean distance and complete linkage. (Heatmaps using other datasets can be found in Figure S2 in File S2.).

In addition, as multiple gene sets can be used to derive similar [35] or identical classification schemas, we evaluated whether breast cancer module scores can be used to predict intrinsic subtype classifications using univariate logistic regression modeling and ROC analysis. Figure 3 shows the heatmap of the area under the receiver operator characteristics curve (AUC) values summarizing the predictive performance of the module scores in GSE1456. As expected, Luminal A subtype was best predicted by upregulation of the estrogen signaling module 1-ER, (AUC = 0.926), with a sensitivity of 66% and a specificity of 95% at the Youden optimal threshold. Luminal B subtype was best predicted by downregulation of the developmental/basal module 2-Dev/Basal, (AUC = 0.88), or upregulation of proliferation 11-Prolif (AUC = 0.79) or interferon 3-Immune (AUC = 0.80) modules. Basal subtype was strongly associated with downregulation of the estrogen signaling pathway 1-ER (AUC = 0.96), with a sensitivity of 76% and a specificity of 98% at the Youden optimal threshold, and also significantly associated to upregulation of developmental/basal module 2 (AUC = 0.85) and proliferation module 11 (AUC = 0.83). As expected, Her2 subtype is most strongly predicted by the ERBB2 module 7-ERBB2 (AUC = 0.90), though with a much higher specificity (98%) than sensitivity (53%). Interestingly, Normal subtype is best predicted by upregulation of the hybrid ECM/development module 9-ECM/Dev/Immune (AUC = 0.88), and downregulation of the proliferation module 11-Prolif (AUC = 0.86). These results generalize to all datasets we tested, including METABRIC and GSE21653 (see Figure S2 in File S2 for additional heatmaps, and AUC values for all datasets). As in the gene-based analysis above, immune modules 4/5, histone module 6, and the ECM and stromal modules 8 and 10 are not highly predictive of subtype in any of the datasets that we analyzed.

### Immune Modules are Highly Correlated to Other Published Signatures

Since immune and ECM module genes or expression did not seem to be strongly correlated to intrinsic subtype or to the 70- and 21-gene prognostic signatures, we were curious as to whether these modules capture the same or different information from previously published immune or ECM signatures. Immune signatures under consideration include the STAT1 immune cluster [19], the IR-7 immune ER- prognostic signature [20], the IFN interferon cluster [21], and T cell and B cell surface markers [22]. We investigated relationships between the signatures by calculating Pearson correlation coefficients (r) between signature-module pairs in three datasets – GSE21653, GSE2034, and GSE1456– with median values reported here. Modules 4-Immune and 5-Immune are similar to these published immune signatures, based on covariance of signature scores: module 4-Immune was highly correlated to T cell (r = 0.96) and B cell (r = 0.94) surface markers, and somewhat less so to the STAT1 immune cluster (r = 0.83). Similarly, module 5-Immune was highly correlated to the STAT1 immune cluster (r = 0.94) and to T cell (r = 0.91) and B cell (r = 0.89) surface markers, and somewhat less so to the interferon IFN cluster (r = 0.81) and the IR-7 signature (r = 0.81). Module 3-Immune-IFN appeared to be most similar to the interferon cluster (r = 0.91), with much less shared signal with T cell (r = 0.47) and B cell (r = 0.41) markers. In contrast, the ECM-enriched modules we identified were not as highly correlated to published gene expression signatures classifying ECM components (clusters ECM1, ECM2, ECM3 and ECM4 [36]). Module 10-ECM was only moderately correlated to the ECM3 cluster (r = 0.69), and moderately anti-correlated to the ECM1 cluster (r = −0.57). Finally, we examined correlations between modules and the proliferation signature MS-14 associated with recurrence in hormone receptor positive (HR+) patients [37]. As expected, module 11-Prolif was highly correlated to the proliferation signature MS-14 (r = 0.97). Thus, the immune and proliferation modules are capturing the same information as other published immune and proliferation signatures and markers, but the ECM module appears to be distinct (see Figure S3 in File S2 for the correlation heatmap, and Table S1 in File S2 for the correlation coefficients).

### Tumor Intrinsic vs. Extrinsic Modules

Since the microenvironment is known to play an important role in breast cancer [38], and since most of the gene expression datasets used to derive the modules are from tumor samples containing a mixture of epithelial cells, stroma, and infiltrating immune cells, we attempted to assess which of the modules might be tumor cell-intrinsic (gene co-expression occurring within the actual malignant cells) and which tumor cell-extrinsic (gene co-expression occurring in or dependent on other cells in the tumor microenvironment). To investigate, we compared module score distributions and coherence (the relative co-expression of the genes in each module) in tumors relative to breast cancer cell lines. Specifically, we applied the F-test to compare the variances of module scores in representative breast cancer cell lines (BCCL; see Methods) and a human tumor dataset (GSE21653), and used a t-test to compare Fisher-transformed Pearson correlation coefficients for all pairs of genes in each module between tumor and BCCL datasets. We reasoned that for tumor-extrinsic modules, module scores might be highly variable across tumor samples and that the expression of the genes within a module would be highly coherent but that the module scores would be less variable and the module gene expression would be less coherent in BCCL. For tumor-intrinsic modules, score variability and gene expression coherence would be high across both types of samples. We hypothesized that the immune and ECM modules would be extrinsic, since we expected the signal for these modules to come from tumor infiltrating immune cells and ECM components that are absent in BCCL cultures.

**Figure 4:**
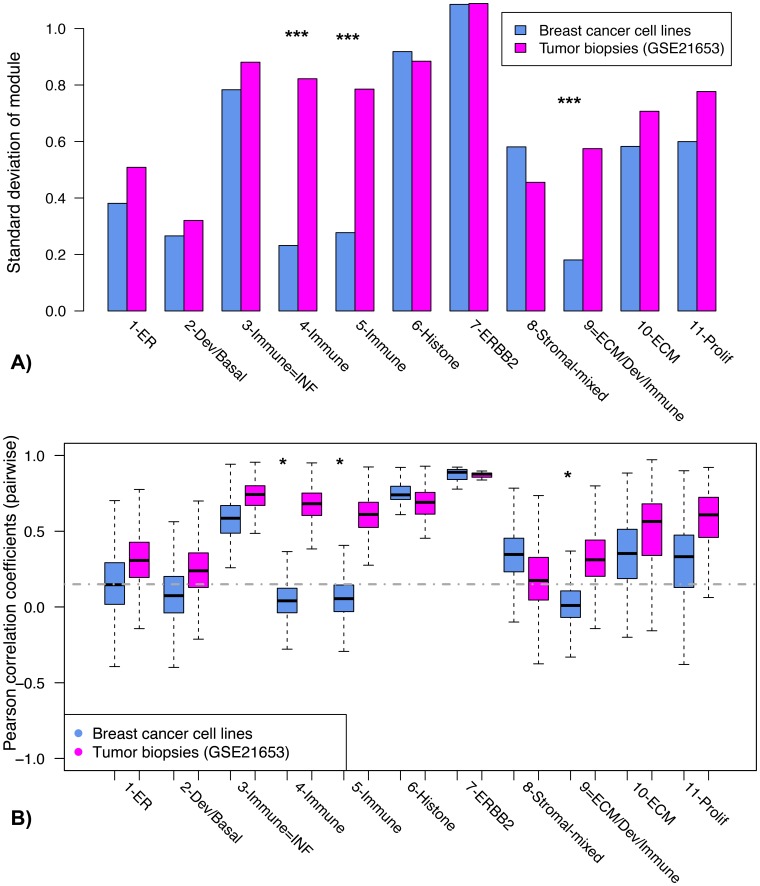
Diversity and coherence of module expression in breast cancer cell lines compared to whole tumors. A) This bar plot compares standard deviations of module scores in representative BCCL (a composite of data from the Sanger, GSK, and Neve et al. datasets, see Methods) and a breast tumor biopsy dataset (GSE21653). *** p<1E-10 (F-test for difference in variance in module score). B) This box plot shows the distributions of Pearson correlation coefficients for all pairs of genes in each module, respectively, for the BCCL and tumor datasets. *Modules 4-Immune, 5-Immune, and 9-ECM/Dev/Immune can be considered tumor-extrinsic, as their constituent genes are uncorrelated in cell lines but highly correlated in patient tumor biopsies (median r>0.35).

We indeed found T cell/B cell immune modules 4-Immune and 5-Immune to be extrinsic, with much more variable module scores in human tumors than in BCCL (p-values <2E-21; Figure 4A) and much more correlated gene expression in breast tumors (mean Fisher-transformed r = 0.85 and 0.73, respectively) than in the breast cancer cell line panel (mean Fisher r = 0.045 and 0.068, respectively) (Figure 4B). In contrast, the interferon-related immune module 3-Immune-IFN appears to be intrinsic, with equally variable module scores in both tumors and BCCL (p-value 0.16) and correlated gene expression in both tumors (mean Fisher r = 0.97) and cell lines (mean Fisher r = 0.69). The module most highly enriched for ECM-related genes, 10-ECM, was coherent in cell lines (mean Fisher r = 0.38) as well as in breast tumors (mean Fisher r = 0.58), with similar module score variability in both contexts (Figures 4A–B), and thus appears to be tumor cell intrinsic. Module 9-ECM/Dev/Immune, with its mixture of ECM, immune, and developmental genes, also had coherent gene expression in the tumor biopsy dataset (mean Fisher r = 0.36) but not in the breast cancer cell lines (mean Fisher r = 0.027) (Figure 4B), with variable expression in the former but not the latter (p-value<9E-34; Figure 4A), and thus appears tumor-extrinsic. To assess whether the pattern we observed in GSE21653 applies more generally, we analyzed several additional data sets (GSE1456, GSE3494, GSE2034) and concluded that the intrinsic/extrinsic classifications generalize to all datasets we tested (see Figure S4 in File S2). Taken together, these data suggest that BCCL reflect much of the gene expression diversity between tumors with the exception of the tumor cell-extrinsic modules 4-Immune, 5-Immune and 9-ECM/Dev/Immune.

We also used t-tests to compare module expression levels in microdissected tumor epithelium and stroma (GSE5847). As expected, ECM modules 8–10 have significantly higher mean expression levels in stroma compared to epithelium (see Figure S5 in File S2). Expression of immune modules 3–5, however, did not differ in the two compartments. These results suggest either immune contamination in the epithelial compartment, or immune-specific signaling of epithelial cells that occurs only in a native (not cell line) micro-environment.

### Low Expression of Immune Modules in a High-proliferation Background Predicts Poor Prognosis

Historically, the development of effective treatments for breast cancer, such as Herceptin for HER2+ breast cancer and endocrine therapy for ER+ breast cancer, has been driven by observations of recurrent molecular aberrations in tumors that are associated with differential patient outcomes. Our breast cancer co-expression modules were derived independently of clinical data, and thus we wondered whether any modules might have prognostic significance. To investigate, we scored a previously published [31], pooled dataset of 683 adjuvant untreated node-negative patients from datasets GSE2034, GSE5327, GSE7390 and NKI295 for module expression, and performed univariate and multivariate Cox Proportional Hazards survival modeling with and without adjustment for receptor status and the proliferation module 11-Prolif. All p-values were adjusted for multiple testing using the Benjamini-Hochberg (BH) method; associations with a BH p-value<0.05 were considered significant.

The very strongest association to recurrence was high proliferation (see Table S2 in File S2 for all p-values). Consistent with many prior studies, we found that high expression of the proliferation module 11-Prolif was significantly associated with decreased RFS, and high expression of the estrogen signaling module 1-ER was significantly associated with increased RFS. Both associations retained statistical significance even after adjustment for ER and Her2 status, suggesting that the ER module 1-ER might be capturing information on recurrence risk beyond that encoded in ER status.

**Figure 5:**
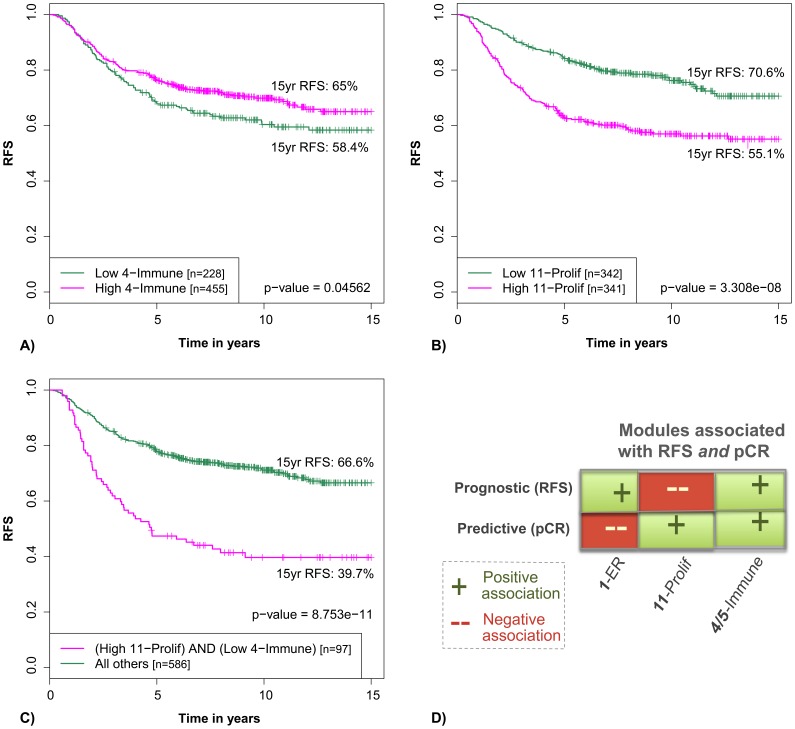
Recurrence free survival of chemotherapy naïve patients with highly proliferating tumors depends on immune module activation. Kaplan-Meier analysis shows that patients with high 11-Proliferation expression AND low 4-Immune expression have poorer outcomes than patients with low 11-Proliferation OR high 4-Immune expression (C). To demonstrate how dividing the patients according to the activity of both modules increases sensitivity to detect patients with poorer outcome, we include K-M plots of RFS as a univariate function of 4-Immune (A) and 11-Prolif (B). Module activity was dichotomized using the median for 11-Prolif and the lower tertile for 4-Immune. D) Immune modules are an exception to a Simpson’s paradox in breast cancer that some of the same features that are associated with poor outcome are also associated with superior response to chemotherapy (high PCR rate). Modules 11-Prolif and 1-ER both conform to this paradox, as high 11-Prolif is associated with a good response to chemotherapy but a poor outcome, whereas high 1-ER is associated with good outcome but a poor response to chemotherapy. Immune modules 4/5-Immune are an exception to this paradox, as they are associated with a good outcome without chemotherapy *and* a good response to chemotherapy in treated populations.

The more interesting associations were immune and stromal. The stromal module 9-ECM/Dev/Immune was significantly associated with increased RFS in univariate and receptor-adjusted analysis, though not after adjustment for proliferation, suggesting that the stromal milieu represented by this module is associated with less proliferative tumors. The B cell/T cell module 4-Immune was associated with decreased RFS in univariate analysis (Figure 5A), in multivariate analysis adjusted for ER and HER2 status, and in multivariate analysis adjusted for receptor status and proliferation. It was significant overall, and in ER positive and ER negative subsets (see Figure S6 in File S2 for survival plots).

In a multivariate Cox regression analysis, the combination of T cell/B cell immune module down-regulation and proliferation module upregulation associated with decreased RFS in these chemo-naïve patients (more so than immune down-regulation or proliferation up-regulation alone), suggesting that cancers with a high proliferation rate in the absence of an activated immune system are prone to recur (Figure 5A–C). These results demonstrate that co-expression modules, especially those reflecting proliferation, immune, and stromal/developmental pathways, associate with differential survival of patients even after adjustment for clinical variables.

### Immune Modules are Associated with Response to Chemotherapy *and* to a Good Prognosis without Chemotherapy

A paradox in breast cancer is that some of the same features that are associated with poor long term survival, such as high grade and ER negativity, are also associated with superior response to chemotherapy (high pCR rate) in the neoadjuvant setting; conversely, features associated with better long term survival, such as Luminal A classification and low grade, are associated with inferior chemo-sensitivity (low pCR rate) [39]. Given this paradox and its likely relevance to optimizing treatment strategies, we were interested in investigating whether modules associated with the prognosis of chemo-naive patients were the same or different from those predicting response to chemotherapy, and for those that overlap, in determining the direction of association.

To this end, we constructed logistic regression models of pathologic complete response (pCR) to neoadjuvant chemotherapy as a function of module scores in groups of patients with gene expression data from pre-treatment biopsies (GSE22093; [40]), and compared the results to the RFS association analyses of adjuvant untreated patients summarized in Table S2 in File S2. For modules associated with the prognosis of chemo-naïve patients *and* with response to chemotherapy, three patterns were observed based on the direction of association (Figure 5D and Table S3 in File S2; significance threshold: BH p-value <0.05). High expression of the estrogen module 1-ER was significantly associated with a good prognosis but a poor response to chemotherapy (positive prognostic, negative predictive). Upregulation of the proliferation module 11-Prolif was significantly associated with a poor prognosis, but a good response to chemotherapy (negative prognostic, positive predictive). These observations are consistent with the prognosis/chemo-response paradox described above. The third pattern we observed, of biomarkers that significantly associate with good prognosis *and* a good response to chemotherapy, is less well established. The cytotoxic T/B cell immune modules 4-Immune and 5-Immune fall in this category, as patients with highly expressed immune modules are more likely to respond well to chemotherapy than those with low immune module expression, *and* are also more likely to have a good prognosis without chemotherapy (positive prognostic, positive predictive). This last category suggests a subpopulation of high immune patients who respond well to chemotherapy but would do well without it.

Notably, in multivariate analysis, the most significant module pairs for predicting pCR were the combined high expression of any of the cytotoxic T/B cell immune modules, 4-Immune especially, with high expression of the proliferation module 11 (AUC = 0.79; Table S4 in File S2), the same pair highlighted in Figure 5 that best predicts RFS in chemo-naïve patients.

### ECM/stromal Modules Associate with Different Sites of Metastasis

**Figure 6:**
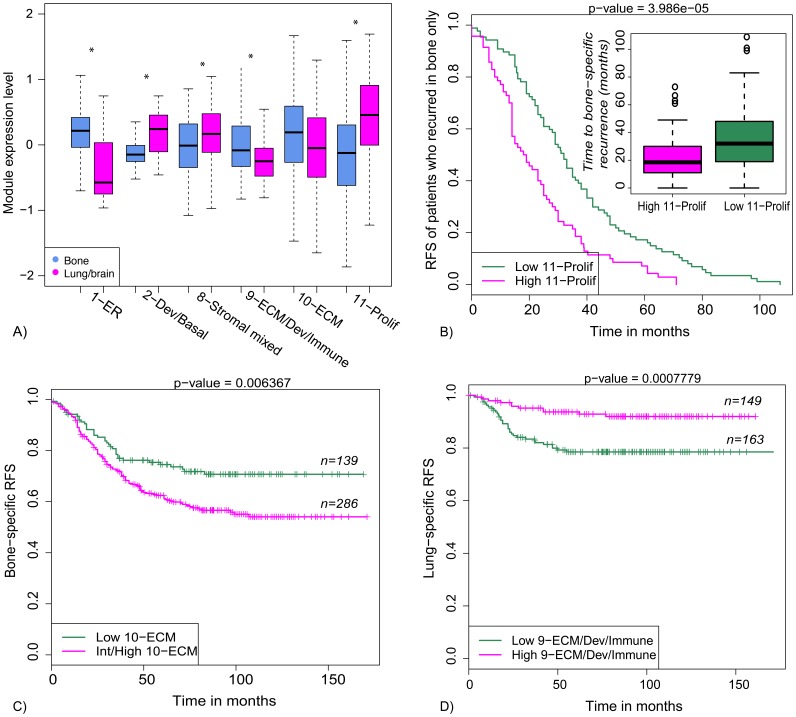
Different organ sites of metastasis are associated with different ECM/stromal modules. A) Boxplot of ECM/stromal module expression in primary tumors that metastasized to bone only vs. lung or brain. Also included are the non-stromal subtype-associated modules with the strongest associations, 1-ER (preferential to bone), and 2-Dev/Basal and 11-Prolif (preferential to viscera). Upregulation of 10-ECM was associated with decreased bone-specific RFS (C), whereas downregulation of 9-ECM/Dev/Immune was associated with decreased lung/brain-specific RFS (D). Upregulation of the proliferation module 11-Prolif was associated with a shorter time to recurrence in bone (B) and lung (Table S6 in File S2), as opposed to 1-ER, which associates with longer times to recurrence to either site (also Table S6). Asterisks in (A) denote statistically significantly different (see Table S5 in File S2 for p-values).

The “seed and soil” hypothesis states that specific organs may harbor metastases from one type of cancer by stimulating their growth better than other types of cancer, in an interaction that is dynamic and reciprocal [41]. Since many of the modules are dominated by ECM or other stromal themes, we wondered whether there might be a relationship between the ECM-related modules and preferred site of metastasis, or progression tempo of disease at different sites. To investigate, we used the clinical site-specific metastasis annotation assembled by Bos and colleagues in their brain metastasis study [42] to assemble a pooled dataset of 572 samples from 3 GEO data sets (GSE2034, GSE2603, GSE12276), pre-processed as described in Methods. In this dataset, there were 261 patients who developed metastases in lung, bone, brain, or multiple sites, and 311 patients who remained metastasis free over the course of their follow up. We asked three questions: first, we used Cox proportional hazards modeling to look for associations between module expression levels and development of metastasis in a specific site. This involved survival analysis of patients who did not recur (n = 311) combined with those who recurred in a specific site, for instance bone only (n = 157; for a total of 468 cases). A second type of analysis concerned site of metastasis among those who recurred. We used logistic regression modeling to assess whether module expression levels were significantly different in patients who developed bone-only metastases (n = 157) as compared patients who developed lung or brain metastases (n = 67). Finally, we performed Cox survival analysis on patients who developed a particular site of metastasis to evaluate the relationship between module expression and time to recurrence. P-values were corrected for multiple testing using the Benjamini-Hochberg method, and multivariate models adjusted for ESR1 and ERBB2 expression were constructed for site-specific RFS and metastasis analyses to assess statistical significance beyond receptor subtype. Results are summarized in Tables S5 and S6 in File S2, and illustrated in Figure 6.

As expected, high 1-ER expression was significantly associated with bone-specific rather than lung- or brain-specific metastases (BH p-value 3.05E-11; Figure 6A), and with longer times to metastasis irrespective of metastatic site (see Table S6 in File S2 for p-values). High expression of 11-Prolif and 2-Basal were associated with visceral metastases and with shorter disease free intervals prior to recurrence (see Figure 6A,B and Tables S5 and S6 in File S2), also consistent with prior publications on the relationship between basal, proliferative cancers and recurrence [1], [2]. The more novel findings concern the stromal modules 8–10, which appear to associate with different sites of metastases despite similar themes and correlated expression patterns (Pearson r = 0.38–0.66). We found that high expression of 10-ECM was associated with decreased bone-specific RFS even after adjustment for ER and ERBB2 (Figure 6C, Table S6 in File S2). In contrast, the stromal module 8-stromal-mixed was associated with decreased lung- and brain- specific RFS and with lung or brain metastases rather than bone metastases in a logistic model (Tables S6, S5 in File S2). Module 9-ECM/Dev/Immune was similar to 1-ER in that it associated with bone-specific rather than visceral metastases (Table S5 in File S2), and with increased lung-specific RFS (Figure 6D), though it did not associate with longer time to recurrence in those patients who suffered recurrence (Table S6 in File S2). Together, these results suggest that the stromal microenviroments represented by modules 8–10 might play a role in the preferred sites of metastasis of breast cancers, possibly relating to the ‘seed and soil’ hypothesis of cancer dissemination.

## Discussion

In this study, we identified 11 breast cancer co-expression modules comprising 958 genes (Table 1), using 72 datasets of publicly available gene expression data from breast cancer patients and breast cancer cell lines (File S1). Each module consists of a block of genes with bimodal expression patterns and highly correlated expression levels in multiple datasets. A caveat of our module identification algorithm, which selected only those genes with bimodal expression patterns, and only those clusters that appear with high fidelity in multiple datasets, is that there may be additional clusters that represent aspects of breast cancer biology that either might be less commonly interrogated by datasets in our compendium or which have a less dramatic effect on gene expression; in addition, the uneven stability results in the partitioning of stromal modules 8 and 10 suggests that analysis of an alternate collection of datasets might have identified somewhat different stromal coexpression clusters. This filtering method, however, allowed us to exclude patterns of gene expression that are private to individual datasets such as technical artifacts, as well as weaker patterns of coordinate gene expression identified in the clustering step. This work is similar in spirit to the study of Bessarabova et al [14], though it differs in the number of independent data sets that were used to derive the modules, in the combined use of human tumor and breast cancer cell line datasets, the methods used to define bimodal gene expression and modularity, and unlike the work reported by Bessarabova et al. our explicit goal was to define common modules across datasets.

**Figure 7:**
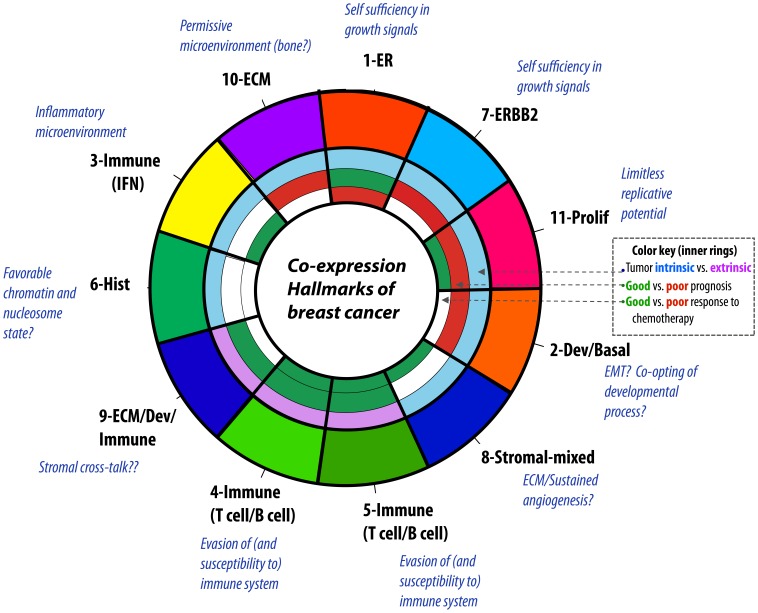
Co-expression module hallmarks of breast cancer. This figure is a model of possible correspondence between co-expression modules and cancer hallmarks, annotated by clinical associations and coherence in cell lines vs. tumors. The inner annotation ring is colored to represent association to chemotherapy response; the 2^nd^ from center represents association to RFS in adjuvant untreated patients and site-specific RFS in a mixed treated/untreated patients; and the 3^rd^ from center classification of intrinsic or extrinsic.

The 11 co-expression modules in breast cancer that we identified represent many of the biological properties and processes that are known to vary between breast tumors and reflect many of the functions implied by the ‘hallmarks of cancer’ (see Figure 7). As expected, in addition to modules involved in estrogen (1-ER) and Her2 signaling (7-ERBB2), we identified a module involved in cell proliferation (11-Prolif) and another module enriched for basal-cell related genes (2-Dev/Basal). Additional tumor-cell extrinsic modules seem related to T cell and B cell immune system activity (4–5-Immune), the importance of which for anti-tumoral activity is increasingly well appreciated. We also identified a number of modules that reflect heterogeneity between breast tumors that are perhaps somewhat more novel. These include a tumor-cell intrinsic immune-related module that is strongly enriched for interferon-related genes (3-Immune IFN), as well as a module consisting exclusively of histones (6-Hist) and three modules of genes enriched in ECM and stromal-related genes (8–10). One can think of these co-expression modules as a data reduction transformation: several tens of thousands of probes representing genes are reduced to a handful of modules representing a higher order organization of genetic regulatory function in breast cancer. Thus, each woman’s tumor can be categorized in terms of the activity levels or subclassifications over each of these modules (e.g., tumor A has downregulated estrogen signaling in a high T cell/B cell immune, highly proliferative background, without high ERBB2 signaling but with a richly upregulated ECM scaffold).

We compared the modules to intrinsic subtype and other well-known prognostic signatures to see if our data driven approach was able to rediscover known aspects of breast cancer heterogeneity, and possibly uncover new themes.

We found that a little over half of PAM50 genes used to evaluate intrinsic subtype were also found in modules, but only a minority of the modules were represented among the PAM50 genes (Table 2): estrogen signaling (1-ER), proliferation (11-Prolif), Her2 signaling (7-ERBB2), and to a small degree the developmental/basal module (2-Dev). Similarly, genes from the 21-gene recurrence score and the 70-gene prognosis signature that overlap module genes are also distributed among these same four modules. Genes from immune modules 3–5, histone module 6-Histone, the mixed development/immune module 9-Dev/Immune, and the ECM modules 10-ECM and 8 are not represented in these signatures. Moreover, these modules were not highly predictive of intrinsic subtype, further suggesting that they might represent additional aspects of breast cancer biology variability and we were thus interested to know if these modules might define clinically significant differences between tumors.

The immune modules, 4-Immune and 5-Immune especially, do however appear to be very well represented by several published immune signatures. Module 4-Immune was highly correlated to T cell and B cell surface markers [22], module 5-Immune to the STAT1 immune cluster [19], and Module 3-Immune to the interferon IFN cluster [21]. In contrast, ECM-enriched modules 9-Dev/ECM/Immune and 10-ECM, both significantly upregulated in stroma relative to epithelium, are not as well represented by the published ECM signatures we evaluated [36]. The proliferation module 11-Prolif is highly correlated (nearly interchangeable) to the proliferation signature MS-14 [37] associated with prognosis of HR+ patients, as well. These results add further evidence to the growing body of work emphasizing the importance of immune signaling and proliferation in breast cancer, and point out that despite the different origins of these signatures, they seem to converge on common signals and resulting classifications.

Since breast cancer progression is known to be influenced by the microenvironment, we tried to assess modules as being tumor cell-intrinsic or tumor cell-extrinsic by comparing module expression in breast cancer cell lines and human tumors. As expected, coordinated differential expression of either of the two B Cell/T cell immune modules was not observed in the breast cancer cell lines, perhaps reflecting the presence of infiltrating immune cells in tumors. One immune module appears to be tumor cell intrinsic, however: the interferon responsive 3-Immune IFN module. The ECM module 10-ECM was similarly intrinsic despite the lack of tumor stroma interactions, while Module 9-ECM/Dev/Immune was extrinsic. These differences are worth investigating further, as they may provide clues as to the nature of the ‘dynamic reciprocity’ between tumor cells and their local ecology and microenvironment [43]. Overall, the analysis of module expression variability and coherence in breast cancer cell lines as compared to tumors suggests that cell lines reflect most of the diversity found between tumors with the exception of modules 4-Immune, 5-Immune and the stromal module 9-ECM/Dev/Immune. Importantly, these ‘extrinsic’ modules are associated with patient outcome, response to standard chemotherapy, or preferential site of metastasis, and thus point to themes that if included might improve in vitro models.

As some of our modules reflect aspects of breast cancer heterogeneity that are currently used for clinical decision making, we expected that these modules would be associated with clinical outcome. We were similarly interested in whether some of our more novel modules might also be clinically important as they might then be a useful source for biomarkers or drug targets discovery.

In univariate analysis, upregulation of the proliferation module (11-Prolif) was significantly associated with recurrence in node negative, adjuvantly untreated patients. Upregulation of T cell/B cell immune modules associated with recurrence free survival, even after adjustment for ER, ERBB2, and proliferation. These immune modules were significant in the population as a whole, and in ER positive and ER negative patient subsets. Another interesting module was the stromal module 9, with its mix of developmental, immune, and ECM genes, which also associated with recurrence free survival.

In multivariate analysis, the most robust pair of modules for predicting recurrence in these chemo-naive patients was the combination of a low Tcell/Bcell immune module score and a high proliferation module score, suggesting that cancers with a high proliferation rate in the absence of an activated immune system are prone to recur.

Our multivariate results are consistent with the winning strategy by the Metagene Attractor team (Cheng, Yang and Anastassiou) in the Sage Bionetworks-DREAM breast cancer prognosis challenge, of applying immune and (LYM) and proliferation (CIN) metagenes derived from an analysis of co-expressed genes in multiple cancer types to successfully predict prognosis in the METABRIC dataset (https://sagesynapse.wordpress.com/category/breast-cancer-challenge/) [44]. These results also suggest that treatment strategies that target proliferation while boosting anti-tumoral immunity might be especially effective for high risk patients.

We also found interesting associations between some of the more novel modules and site specific metastasis. As expected, the estrogen module was associated with bone-specific rather than lung- or brain-specific metastasis, whereas the proliferation and basal modules associated with visceral rather than bone metastasis. In addition, we found that the ECM/stromal modules 8–10 appeared to associate with different sites of metastasis despite similar themes, possibly relating to the ‘seed and soil’ hypothesis of cancer dissemination. Further analysis of these ECM/stromal modules may help identify treatment strategies that target the microenvironment or tumor-microenvironment reciprocity to prevent metastasis.

Finally, in comparing modules associated with response to chemotherapy (pCR vs. not) to those associated with the prognosis of chemotherapy-naive patients, the most common pattern we observed was that of modules associated with good prognosis *or* a good response to chemotherapy (but not both). For instance, high expression of the estrogen module is associated with a good prognosis but a poor response to chemotherapy, whereas upregulation of the proliferation module is associated with a poor prognosis but a good response to chemotherapy. This pattern is consistent with studies suggesting that poor prognosis patients such as those with triple negative disease are more likely to respond to chemotherapy than are good prognosis patients, but that a non-response in these patients likely results in a poor outcome [39]. The third pattern we observed, of biomarkers that associate with good prognosis *and* a good response to chemotherapy, is less recognized. The cytotoxic T/B cell immune modules fall in this category, as patients with highly expressed immune modules were more likely to respond well to chemotherapy than those with low immune module expression, and were also more likely to have a good prognosis without chemotherapy. These results are consistent with numerous publications linking the efficacies of a variety of chemotherapies to anti-tumoral immune responses, and suggest the possibility of a paradox – that in some high-immune patients, the same host processes contributing to an excellent response to chemotherapy might preclude its necessity. This observation further supports a treatment strategy boosting anti-tumoral immunity in low-immunity or check-point blocked patients with highly proliferating tumors, either prior to or in combination with neoadjuvant cytotoxic chemotherapy.

Overall, co-expression modules provide a high-level functional view of breast cancer that complements the ‘cancer hallmarks’ and may form the basis for improved predictors and treatments.

## Methods

### Identifying and Scoring Transcriptional Modules

To identify breast cancer co-expression modules we used the Gene Expression Omnibus (GEO) and several other public sources to assemble a data compendium consisting of 72 public gene expression datasets that had been profiled on U133-generation arrays (U133A, HT-U133A, U133Av2 and U133_Plus2). These datasets were comprised of samples from both human breast tumor and breast cancer cell lines, and the data compendium consisted of a total of 5684 samples (see File S1 for complete list of datasets). Gene-level expression estimates were per dataset obtained using RMA [45] and an EntrezGene-directed CDF [46]. Each dataset was then filtered to the probesets common to the four platforms. Within each dataset, a per array measure of sample quality (avg.z) was derived by first z-score normalizing each gene and then calculating an average expression value per array [47]. The final expression estimates for each gene were the residual of a linear model of measured gene expression as a function of avg.z in each dataset. These quality adjusted expression estimates were used to minimize correlation between gene expression profiles due to differences in array quality. The bimodality of gene expression was scored for each gene within each dataset using MCLUST [48] and the Bimodality Index (BI) [49]. The significance of the observed bimodality was assessed by comparing the observed BI score to BI scores observed in 10,000 random samples of the normal distribution. Each random sample was of the same size as the dataset from which the observed BI score was derived. This empiric p-value was used to derive a Benjamini-Hochberg FDR [50] and genes with a BI FDR <0.05 were considered to have significantly bimodal gene expression in that dataset.

Within each dataset, genes with significantly bimodal gene expression were organized into clusters using a model-based clustering algorithm (MCLUST) and the Bayesian Information Criterion (BIC) to determine the optimal number of clusters [51]. Principal component analysis was performed with the genes in each cluster within the dataset where that cluster was identified. The resulting gene loadings for the first principal component were defined as a metagene for the pattern of gene coexpression in that cluster. The scalar projection of each of the samples in the compendium in the direction of this metagene was used as a score of relative cluster expression. This projection was calculated as the inner product of the normalized gene expression data for each sample and the metagene. The similarity between the gene expression dynamics of each cluster were identified by calculating the pairwise Pearson correlation coefficients (r) between the scores derived for each of the clusters. Clusters with an r >0.7 with at least six other clusters were kept for further analysis under the assumption that these clusters represent frequently observed patterns of dynamic gene expression. The similarity between the expression of these clusters was assessed by hierarchical clustering (Euclidean distance metric, complete linkage clustering) of the Pearson correlation coefficients between clusters and each cluster was assigned to one of eleven modules (Figure 1). To validate the clustering, we used SigClust [23] with 1000 simulations, the “hard thresholding” method reported by Liu et al. for estimating the eigenvalues of the covariance matrix [23], and p-values determined empirically from the simulated null distribution. We also applied the more recently described “soft thresholding” method for estimating the eigenvalues of the covariance matrix used by SigClust [24].

A final step was to identify the common genes within each module: the genes in each of the final modules consisted of the genes that were in >33% of the correlated clusters that contributed to each module. Gene weights for each of the final modules were defined as the first principal component of each gene set across the union set of samples in the datasets that contributed a cluster to the module. New Affymetrix U133 generation datasets were scored for module expression by first RMA and z-score normalizing as described above and then projecting the weight vector for each module (weights in SI_Datasets_Genes) onto that dataset using an inner product. Similarly, for cross-platform application, such as that performed on the Metabric dataset [4] assayed on the Illumina HT-12.v3 platform, we z-score normalized the EntrezGene median-collapsed expression data and projected the module weights onto the module genes represented in the dataset.

### Analyzing Modules for Functional or Pathway Enrichment

To investigate whether co-expression modules contain recognizable functional or regulatory themes, we applied the pathway/functional enrichment analysis software tools DAVID [25] and g:Profiler [26] using multiple testing corrected p-values that control for false discovery, with the whole human genome as background (the default).

### Comparing Modules to Intrinsic Subtype and other Signatures

To determine whether any of the modules we identified were related to clinical breast cancer biomarkers, we calculated the overlap between module genes and the PAM50 intrinsic subtype gene set [1], [32], the NKI70 MammaPrint® gene set [33], and the 21 genes used in OncotypeDX® [34]. Since different gene sets can be used to derive an identical classification schema, we also fit univariate logistic regression models relating intrinsic subtype assignments to module scores in GSE1456, GSE21653, and METABRIC, and then performed ROC analysis on these models to calculate an AUC estimate of how well each individual module is able to predict each subtype. For comparison of modules to other previously published signatures, pretreatment biopsies in GSE21653, GSE1456, and GSE2034 were scored for expression of the STAT1 immune cluster [19], the IR-7 immune signature [20], the IFN interferon cluster [21], the proliferation signature MS-14 [37], and for subsets of T cell and B cell surface markers [22] by calculating the mean expression levels of signature genes weighted by +1 or −1 according to direction of association with RFS as previously described [31]; ECM1-4 cluster scores were calculated as the Pearson correlations between expression of the genes in the published ECM signature and the four ECM centroids, respectively [36]. Pearson correlation coefficients (r) between the module and signature scores were calculated to assess relatedness.

### Comparing Tumor Cell-intrinsic to Tumor Cell-extrinsic Co-expression

To compare co-expression of genes within a module as well as module score variability in breast cancer cell lines (BCCL) and human breast tumor biopsies, we collected Affymetrix gene expression data for cell lines in the Sanger (http://www.broadinstitute.org/cgi-bin/cancer/publications/pub_paper.cgi?mode=view&paper_id=189), GSK (https://array.nci.nih.gov/caarray/project/woost-00041/), and Neve et al. [18] datasets, RMA normalized with quality adjustment as described above, and eliminated redundancy by combining highly correlated cell line samples (r>0.9) with the same name, resulting in a BCCL dataset of 111 cell line samples. Modules in BCCLs were compared to those in human breast tumor biopsies (GSE1456, GSE21653, GSE2034, and GSE3494) by calculating Pearson correlation coefficients for all pairs of genes in each module, respectively, for the two datasets, and by applying a Student’s t-test to the Fisher-transformed correlation coefficients to test for differences in mean correlation levels. Modules with uncorrelated gene expression in BCCL (mean r <<0.1) but correlated gene expression in tumors (median r >0.35) were considered extrinsic. We also used the F-test to compare the variances of the scores from each module in the tumor and BCCL datasets, and applied a t-test to assess differences in module expression in tumor epithelium and stroma (GSE5847; [52]).

### Analyzing Modules for Association with RFS, Chemo-response, and Site-specific Metastases

To assess associations between module scores and breast cancer prognosis, we scored a previously published [31], pooled dataset of 683 adjuvant untreated node-negative patients from datasets GSE2034, GSE5327, GSE7390 and NKI295 for module expression, and performed univariate and multivariate Cox Proportional Hazards survival modeling with and without adjustment for receptor status and proliferation (11-Prolif), using the Survival software package [53] in R. As previously described [31], the processed data from each source was mean-centered independently, mean-collapsed by gene symbol, and the 10,219 unique genes common to all platforms combined using distance weighted discrimination (DWD). To assess the association between module expression and chemotherapy response (GSE22093), we constructed logistic regression models of pathologic complete response (pCR) as a function of module scores followed by ROC analysis using functions from EPICALC and STATS software packages in R [54]. For our analysis of site specific metastasis, we used the clinical site-specific metastasis annotation assembled by Bos and colleagues in their brain metastasis study [42] to assemble a pooled dataset of 572 samples from 3 GEO data sets (GSE2034, GSE2603, GSE12276). These samples were preprocessed by: 1) RMA normalizing samples run on the same platform together, 2) merging datasets by probe id, using the annotation from GEO, and 3) ComBat batch adjusting to combine datasets, prior to scoring them for module expression. We used Cox proportional hazards modeling with and without adjustment for ER and ERBB2 expression to analyze for associations between module expression and site-specific RFS, and logistic regression modeling to assess whether module expression levels were significantly different in patients who developed bone-only metastases as compared to patients who developed lung or brain metastases. P-values were adjusted for multiple testing using Benjamini-Hochberg method [50], and all calculations were performed in the R computing environment [54].

## Supporting Information

File S1 This supplementary file contains a complete list of the datasets used to define the modules, as well as the genes in each module and their associated weights.
(XLS)

File S2 This file contains six supplementary figures and six supplementary tables, as follows: **Figure S1. Examples of the coordinate differential expression of module genes in different breast cancer datasets.** Descriptions of these datasets can be found in file S1. Clustering was performed with Euclidean distance and complete linkage. **Figure S2. Subtype-module relationships are consistent in multiple datasets.** Heatmaps in (A) and (B) show hierarchically clustered AUC scores summarizing how well each intrinsic subtype can be predicted by each coexpression module score. Red denotes high positive predictive value (AUC → 1), green high negative predictive value (AUC → 0), and black a non-informative relationship (AUC≈0.5). Clustering was performed using Euclidean distance and complete linkage. (C) This table shows the mean values of each module in each intrinsic subtype for all three datasets analyzed (GSE21653, METABRIC, and GSE1456), along with AUC values. **Figure S3. Module-signature correlation heatmap.** A correlation heatmap showing the median Pearson correlation coefficient between each module and each published signature, using datasets GSE1456, GSE21653, and GSE2034 (see Table S1 in File S2 for coefficients). Clustering of the correlation coefficients was performed using Euclidean distance and complete linkage. **Figure S4. Intrinsic/extrinsic classifications are consistent in multiple datasets.** (B,D,F) These bar plots compares standard deviations of module scores in representative BCCL (a composite of data from the Sanger, GSK, and Neve et al. datasets, see Methods) and a human breast tumor dataset. *** p<1E-10 (F-test for difference in variance in module score). (A,C,E) These box plots show the distributions of Pearson correlation coefficients for all pairs of genes in each module, respectively, for the BCCL and tumor datasets. ***Modules 4-Immune, 5-Immune, and 9-ECM/Dev/Immune can be considered tumor-extrinsic, as their constituent genes are uncorrelated in BCCLs but highly correlated in human tumor biopsies in all datasets tested (median r>0.35). Datasets: GSE21653 (Figure 4), GSE1456, GSE2034, GSE3494. **Figure S5. Module expression in microdissected tumor stroma vs. epithelium.** We used the dataset GSE5847 to compare module expression levels in micro-dissected tumor epithelium and stroma. Only ECM/stromal modules 8–10 had significantly different expression levels (BH p-value <0.05). **Figure S6. Upregulation of a T cell/B cell immune module was associated with RFS in ER+ and ER- subsets.** These Kaplan-Meier plots show that T cell/B cell immune module 5-immune is significantly associated with RFS in ER+ and ER- patient subsets in our dataset of 683 node-negative adjuvantly untreated cases. Module expression was dichotomized at the median. **Table S1.** Pearson coefficients (r) for module-signature pairs, from multiple datasets. **Table S2.** Recurrence free survival analysis of the pooled prognostic dataset of 683 node-negative adjuvant untreated cases. **Table S3.** Associations between module expression and pCR. **Table S4.** Associations between module pairs and pCR. **Table S5.** Site of metastasis analysis. **Table S6.** Site-specific RFS analysis.
(PDF)
