# Supplementary material for: Gene Co-Expression Modules as Clinically Relevant Hallmarks of Breast Cancer Diversity
Source: PLoS One. 2014 Feb 7;9(2):e88309. doi: 10.1371/journal.pone.0088309 (PMC3917875; doi:10.1371/journal.pone.0088309)
Supplement: File S2 — This file contains six supplementary figures and six supplementary tables, as follows: Figure S1. Examples of the coordinate differential expression of module genes in different breast cancer datasets. Descriptions of these datasets can be found in file S1. Clustering was performed with Euclidean distance and complete linkage. Figure S2. Subtype-module relationships are consistent in multiple datasets. Heatmaps in (A) and (B) show hierarchically clustered AUC scores summarizing how well each intrinsic subtype can be predicted by each coexpression module score. Red denotes high positive predictive value (AUC → 1), green high negative predictive value (AUC → 0), and black a non-informative relationship (AUC≈0.5). Clustering was performed using Euclidean distance and complete linkage. (C) This table shows the mean values of each module in each intrinsic subtype for all three datasets analyzed (GSE21653, METABRIC, and GSE1456), along with AUC values. Figure S3. Module-signature correlation heatmap. A correlation heatmap showing the median Pearson correlation coefficient between each module and each published signature, using datasets GSE1456, GSE21653, and GSE2034 (see Table S1 in File S2 for coefficients). Clustering of the correlation coefficients was performed using Euclidean distance and complete linkage. Figure S4. Intrinsic/extrinsic classifications are consistent in multiple datasets. (B,D,F) These bar plots compares standard deviations of module scores in representative BCCL (a composite of data from the Sanger, GSK, and Neve et al. datasets, see Methods) and a human breast tumor dataset. *** p<1E-10 (F-test for difference in variance in module score). (A,C,E) These box plots show the distributions of Pearson correlation coefficients for all pairs of genes in each module, respectively, for the BCCL and tumor datasets. ***Modules 4-Immune, 5-Immune, and 9-ECM/Dev/Immune can be considered tumor-extrinsic, as their constituent genes are uncorrelated in BCCLs but hi [file pone.0088309.s002.pdf]

Supplemental figures and tables

for PLoS ONE paper:
Gene co-expression modules as clinically relevant hallmarks of breast cancer diversity
by Denise M. Wolf, Marc E. Lenburg, Christina Yau, Aaron Boudreau, Laura J. van 't Veer

Table of contents

Figure S1: Gene clusters in different datasets
Figure S2: Subtype-module relationships A,B heatmap; C data table
Figure S3: Module-signature correlation heatmap
Figure S4: Intrinsic/extrinsic in other datasets
Figure S5: Module expression in stroma vs. epithelium
Figure S6: Immune module stratified survival plot in ER+ and ER- subsets

Table S1: Module-signature correlation coefficients
Table S2: RFS of adjuvantly untreated pooled dataset
Table S3: pCR logistic regression univariate
Table S4: pCR logistic regression bivariate
Table S5: Site of metastasis analysis
Table S6: Site specific RFS analysis

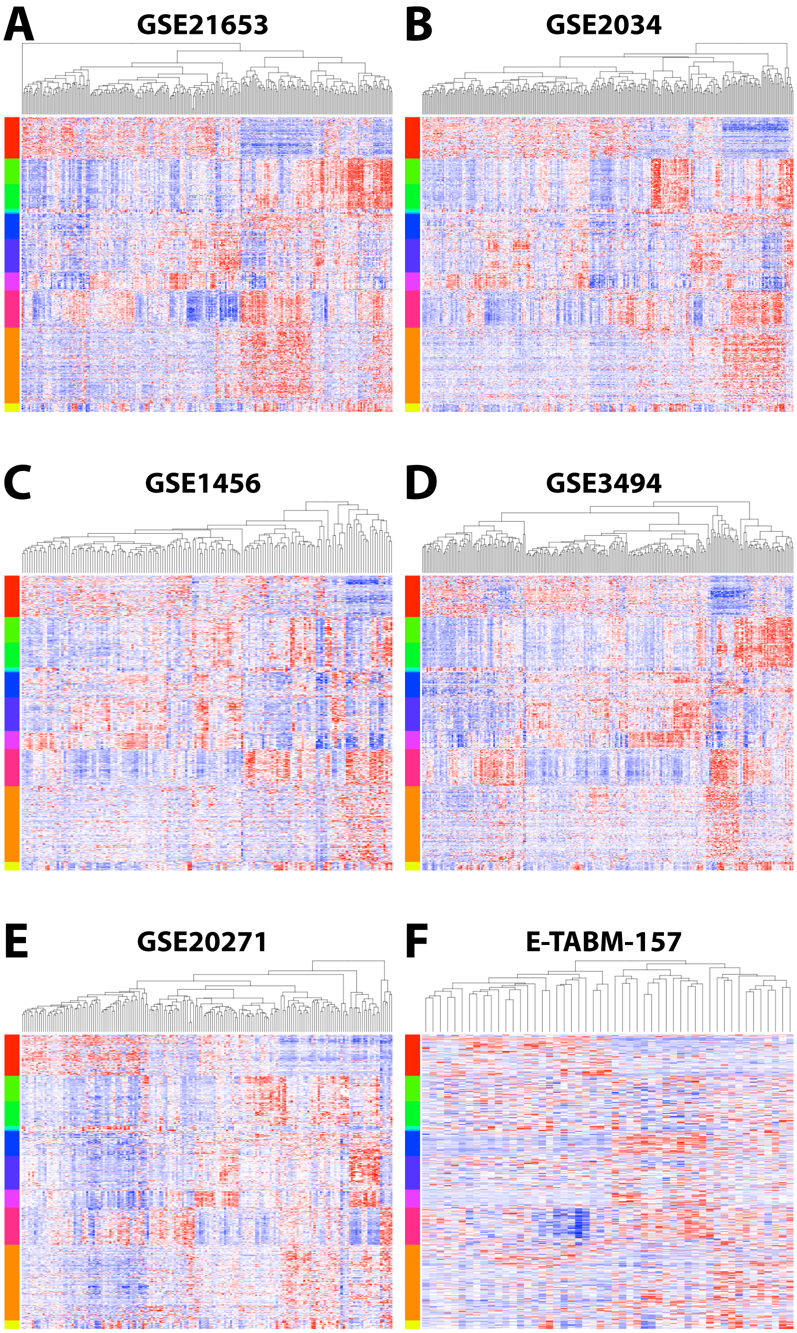

Figure S1. Examples of the coordinate differential expression of module genes in different breast cancer datasets. Descriptions of these datasets can be found in the supplemental information file SI_datasets_genes. Clustering was performed with Euclidean distance and complete linkage.

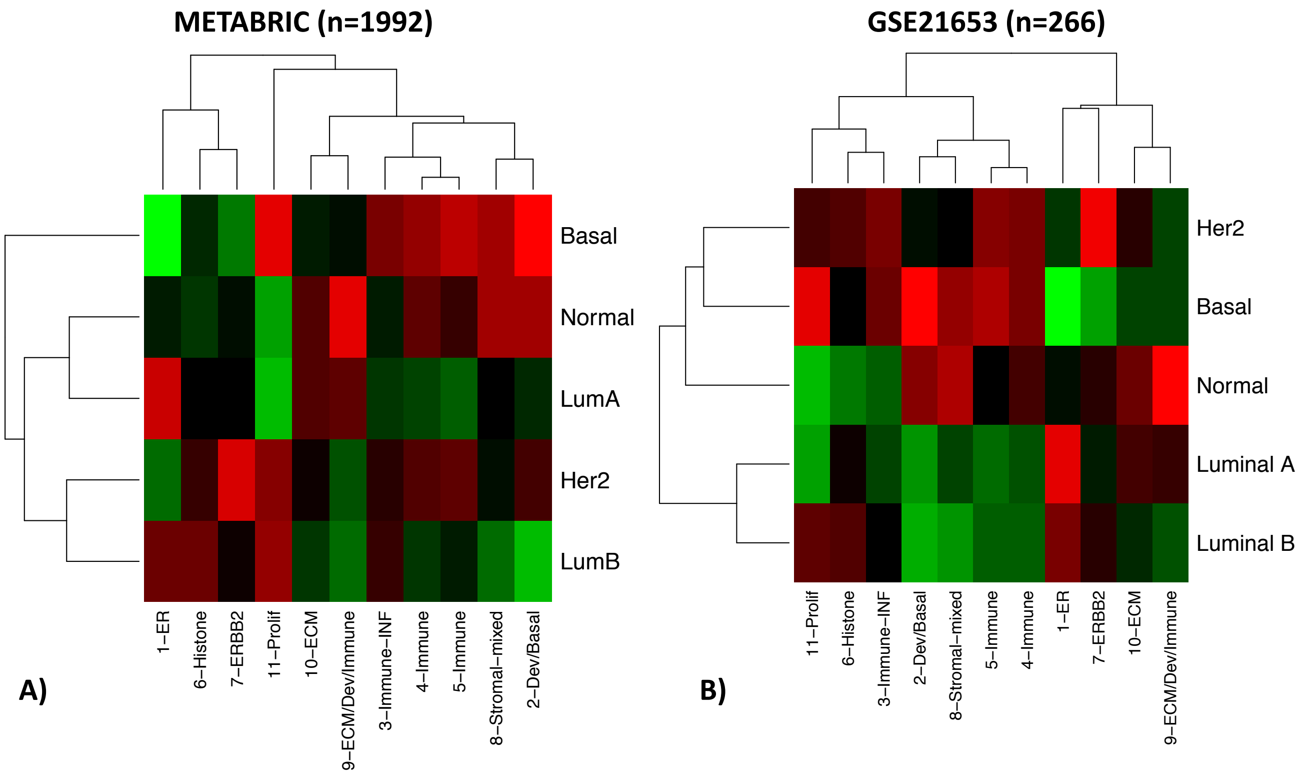

METABRIC dataset (N=1992) Non-AFFY platform

	LumA:Mean	LumA:AUC	LumB:Mean	LumB:AUC	Basal:Mean	Basal:AUC	Her2:Mean	Her2:AUC	Normal:Mean	Normal:AUC
1-ER	0.2762	0.7993	0.1893	0.6462	-0.7256	0.9780	-0.2063	0.7225	-0.0411	0.5730
2-Dev/Basal	-0.0913	0.6053	-0.1952	0.8498	0.4309	0.9132	0.0135	0.5847	0.1584	0.7526
3-Immune-INF	-0.2445	0.6306	0.1149	0.5582	0.4134	0.6732	0.0637	0.5344	-0.2411	0.5872
4-Immune	-0.2440	0.6377	-0.2021	0.6131	0.5010	0.7204	0.2640	0.6055	0.2605	0.6223
5-Immune	-0.2923	0.6835	-0.1488	0.5807	0.6046	0.7814	0.2699	0.6239	0.0906	0.5593
6-Histone	-0.0638	0.5361	0.2829	0.6424	-0.2132	0.5904	0.1545	0.5601	-0.2850	0.6258
7-ERBB2	-0.1792	0.4675	-0.0982	0.5148	-0.4058	0.7438	1.4570	0.8249	-0.1738	0.5556
8-Stromal-mixed	-0.0357	0.5353	-0.2558	0.7247	0.3366	0.7410	-0.0830	0.5591	0.3626	0.7487
9-ECM/Dev/Immu	0.0865	0.6270	-0.2793	0.7067	-0.0906	0.5474	-0.2724	0.6594	0.7112	0.8523
10-ECM	0.1384	0.5951	-0.2144	0.6158	-0.1107	0.5697	-0.0364	0.5134	0.2600	0.6140
11-Prolif	-0.5123	0.8437	0.3069	0.7125	0.7631	0.8544	0.3109	0.6857	-0.6765	0.8156

GSE21653 dataset (N=266)

	Lum A:Mean	LumA:AUC	LumB:Mean	LumB:AUC	Basal:Mean	Basal:AUC	Her2:Mean	Her2:AUC	Normal:Mean	Normal:AUC
1-ER	0.4363	0.8833	0.2937	0.6848	-0.6652	0.9870	-0.1038	0.6159	-0.0290	0.5516
2-Dev/Basal	-0.2069	0.7778	-0.2723	0.8306	0.3705	0.9305	-0.0154	0.4487	0.1496	0.7077
3-Immune-INF	-0.2660	0.6337	-0.0775	0.5250	0.3672	0.6752	0.5784	0.6937	-0.4810	0.6838
4-Immune	-0.3136	0.6730	-0.3955	0.6824	0.4204	0.6986	0.4564	0.7001	0.1658	0.5957
5-Immune	-0.3582	0.7075	-0.4173	0.6877	0.5707	0.7851	0.5206	0.7257	-0.1024	0.5191
6-Histone	-0.0041	0.5031	0.3141	0.6270	-0.0618	0.5307	0.3832	0.6197	-0.6754	0.7266
7-ERBB2	-0.1051	0.4313	0.0067	0.5572	-0.4934	0.8002	1.8315	0.9039	0.0714	0.5383
8-Stromal-mixed	-0.1175	0.6321	-0.3874	0.7912	0.2599	0.7376	-0.0318	0.5226	0.3691	0.7772
9-ECM/Dev/Immu	0.0587	0.5786	-0.2624	0.6540	-0.1976	0.6297	-0.2435	0.6348	0.9757	0.9424
10-ECM	0.1802	0.6085	-0.2199	0.5923	-0.2322	0.6524	0.1071	0.5591	0.3305	0.6617
11-Prolif	-0.5270	0.8092	0.2907	0.6410	0.7143	0.8754	0.1992	0.5851	-0.8862	0.8638

GSE1456 dataset (N=159)

	Lum A:Mean	Lum A:AUC	Lum B:Mean	LumB:AUC	Basal:Mean	Basal:AUC	HER2:Mean	HER2:AUC	Normal:Mean	Normal:AUC
1-ER	0.4001	0.9265	-0.0096	0.5633	-0.6778	0.9618	-0.2068	0.7329	0.0884	0.5448
2-Dev/Basal	-0.1544	0.7314	-0.2571	0.8827	0.3452	0.8510	0.0717	0.6375	0.1095	0.7244
3-Immune-INF	-0.0132	0.4897	0.7914	0.8002	0.2370	0.6015	0.2477	0.6222	-0.6030	0.7924
4-Immune	-0.3429	0.6855	0.5621	0.7068	0.0293	0.5096	0.2597	0.6264	-0.0793	0.5067
5-Immune	-0.3086	0.6485	0.5956	0.7420	0.2363	0.6015	0.3273	0.6732	-0.2567	0.6345
6-Histone	0.2965	0.6346	0.5055	0.6563	-0.4183	0.6510	0.1972	0.5741	-0.4448	0.6874
7-ERBB2	-0.2053	0.4468	0.1844	0.5154	-0.6000	0.7779	2.1573	0.9093	-0.2026	0.5195
8-Stromal-mixed	-0.1566	0.6310	-0.2727	0.7260	0.2093	0.6113	-0.0952	0.5847	0.2005	0.7120
9-ECM/Dev/Immu	-0.2140	0.6220	-0.4162	0.7558	-0.1215	0.5928	-0.3706	0.6880	0.6119	0.8824
10-ECM	-0.0884	0.5391	-0.2955	0.6682	-0.3289	0.6525	-0.0049	0.5079	0.2983	0.6631
11-Prolif	-0.2804	0.6120	0.5857	0.7906	0.8434	0.8316	0.3662	0.6921	-0.6302	0.8562

C)

Figure S2: Subtype-module relationships are consistent in multiple datasets. Heatmaps in (A) and (B) show hierarchically clustered AUC scores summarizing how well each intrinsic subtype can be predicted by each coexpression module score. Red denotes high positive predictive value (AUC → 1), green high negative predictive value (AUC → 0), and black a non-informative relationship (AUC≈0.5). Clustering was performed using Euclidean distance and complete linkage. (C) This table shows the mean values of each module in each intrinsic subtype for all three datasets analyzed (GSE21653, METABRIC, and GSE1456), along with AUC values.

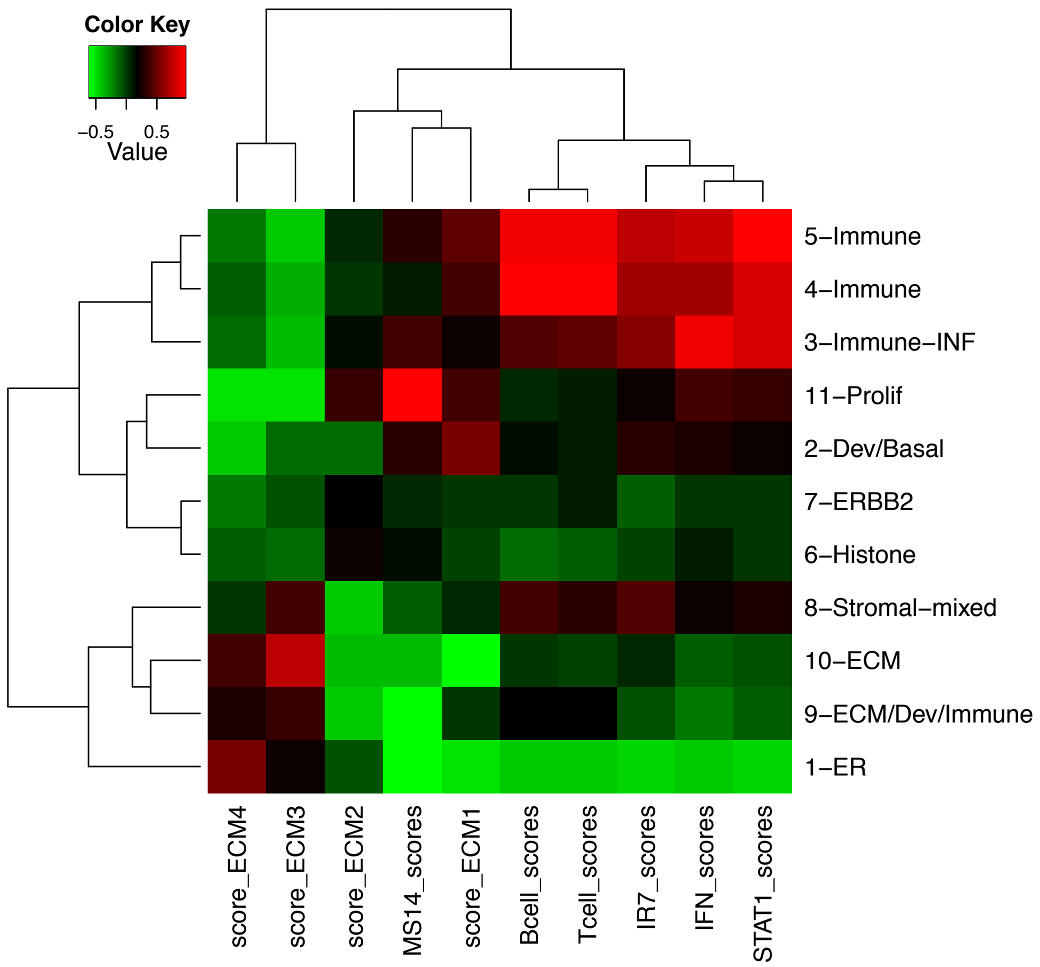

Figure S3: Module-signature correlation heatmap. This correlation heatmap shows the median Pearson correlation coefficient between each module and each published signature, using datasets GSE1456, GSE21653, and GSE2034 (see Table S1 for coefficients). Clustering of the correlation coefficients was performed using Euclidean distance and complete linkage.

	1-ER	2-Dev/ Basal	3-Immune-INF	4-Immune	5-Immune	6-Histone	7-ERBB2	8-Stromal-mixed	9-ECM/Dev/ Immune	10-ECM	11-Prolif
	**median r (min r, max r)**	**median r (min r, max r)**	**median r (min r, max r)**	**median r (min r, max r)**	**median r (min r, max r)**	**median r (min r, max r)**	**median r (min r, max r)**	**median r (min r, max r)**	**median r (min r, max r)**	**median r (min r, max r)**	**median r (min r, max r)**
**STAT1**	-0.452 (-0.518,-0.45)	0.227 (0.086,0.272)	0.818 (0.793,0.849)	0.829 (0.806,0.856)	0.936 (0.924,0.94)	0.0317 (-0.0307,0.0825)	0.009 (-0.1,0.0419)	0.259 (0.216,0.371)	-0.0891 (-0.107,-0.0678)	-0.0735 (-0.199,0.0176)	0.325 (0.302,0.333)
**IR7**	-0.452 (-0.512,-0.402)	0.308 (0.219,0.37)	0.597 (0.47,0.636)	0.654 (0.583,0.695)	0.753 (0.7,0.811)	-0.0243 (-0.027,-0.0135)	-0.0825 (-0.129,0.0297)	0.405 (0.389,0.524)	-0.0755 (-0.104,-0.0351)	0.0654 (0.0179,0.113)	0.226 (0.207,0.263)
**Tcell**	-0.409 (-0.414,-0.376)	0.0885 (0.058,0.25)	0.475 (0.42,0.574)	0.964 (0.956,0.966)	0.911 (0.905,0.938)	-0.0971 (-0.112,-0.0434)	0.0923 (-0.0048,0.102)	0.283 (0.253,0.307)	0.176 (0.0881,0.216)	-0.016 (-0.0424,-0.0028)	0.0844 (0.0611,0.147)
**Bcell**	-0.412 (-0.432,-0.371)	0.147 (0.13,0.251)	0.413 (0.359,0.518)	0.935 (0.926,0.942)	0.895 (0.882,0.898)	-0.143 (-0.183,-0.0814)	0.0262 (-0.0669,0.0282)	0.381 (0.379,0.409)	0.192 (0.192,0.269)	0.0117 (0.004,0.071)	0.0469 (0.0011,0.0482)
**MS14**	-0.581 (-0.597,-0.568)	0.317 (0.234,0.326)	0.369 (0.318,0.385)	0.0869 (0.0864,0.145)	0.291 (0.272,0.317)	0.138 (0.0942,0.272)	0.0463 (-0.116,0.225)	-0.0849 (-0.277,-0.0543)	-0.586 (-0.603,-0.496)	-0.372 (-0.43,-0.363)	0.971 (0.963,0.974)
**IFN**	-0.416 (-0.485,-0.411)	0.254 (0.126,0.289)	0.907 (0.9,0.917)	0.658 (0.642,0.713)	0.809 (0.794,0.822)	0.115 (0.0135,0.128)	0.0196 (-0.0695,0.0452)	0.21 (0.155,0.378)	-0.177 (-0.204,-0.158)	-0.113 (-0.238,0.0375)	0.373 (0.334,0.378)
**ECM1**	-0.501 (-0.594,-0.4)	0.562 (0.517,0.652)	0.231 (0.187,0.288)	0.374 (0.334,0.397)	0.455 (0.379,0.462)	-0.0139 (-0.0593,0.0277)	0.0151 (-0.144,0.122)	0.0499 (-0.0513,0.113)	0.0167 (-0.0048,0.0447)	-0.608 (-0.646,-0.572)	0.385 (0.369,0.443)
**ECM2**	-0.0446 (-0.073,0.049)	-0.126 (-0.199,-0.114)	0.135 (-0.0717,0.229)	0.0089 (-0.0976,0.0229)	0.077 (-0.114,0.0835)	0.225 (0.104,0.255)	0.2 (0.0512,0.213)	-0.412 (-0.448,-0.389)	-0.429 (-0.471,-0.255)	-0.401 (-0.422,-0.259)	0.332 (0.138,0.364)
**ECM3**	0.227 (0.202,0.28)	-0.124 (-0.179,-0.079)	-0.383 (-0.468,-0.274)	-0.344 (-0.346,-0.331)	-0.424 (-0.432,-0.412)	-0.162 (-0.226,-0.143)	-0.0694 (-0.142,0.141)	0.367 (0.267,0.387)	0.333 (0.273,0.344)	0.762 (0.688,0.765)	-0.488 (-0.501,-0.483)
**ECM4**	0.533 (0.433,0.571)	-0.41 (-0.423,-0.297)	-0.126 (-0.19,0.0129)	-0.118 (-0.129,-0.088)	-0.191 (-0.254,-0.133)	-0.0936 (-0.13,-0.0359)	-0.201 (-0.324,-0.0238)	0.0275 (0.0107,0.224)	0.281 (0.186,0.327)	0.392 (0.315,0.497)	-0.5 (-0.52,-0.423)

Table S1: Tabulated Pearson coefficients (r) for module-signature pairs, from datasets GSE21653, GSE2034, and GSE1456. Signatures under consideration include the STAT1 immune cluster [19], the IR-7 immune ER- prognostic signature [20], the IFN interferon cluster [21], T cell and B cell surface markers [22], ECM components (clusters ECM1, ECM2, ECM3 and ECM4 [36]) and cell proliferation [37].

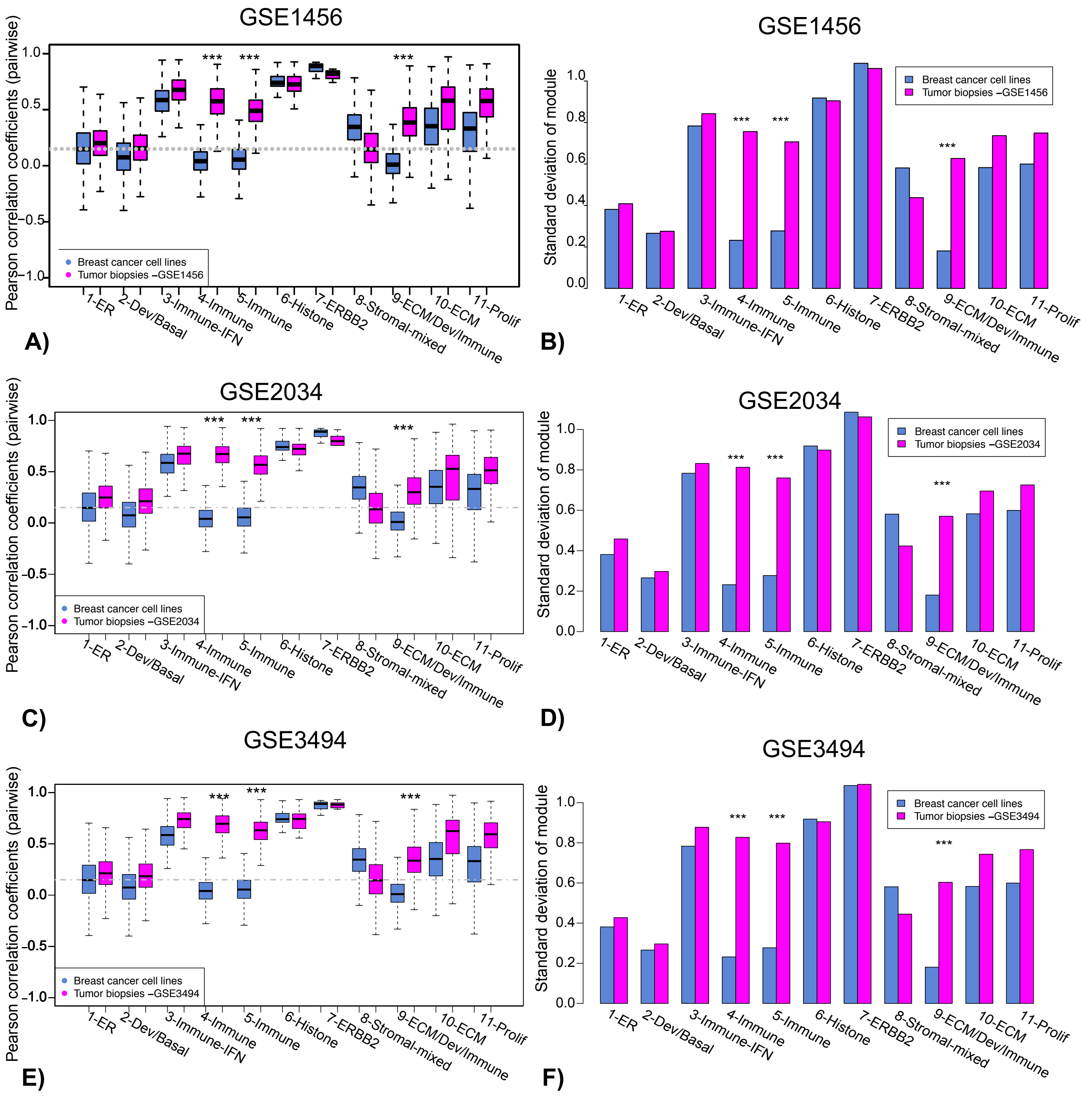

Figure S4: Intrinsic/extrinsic classifications are consistent in multiple datasets. (B,D,F) These bar plots compare standard deviations of module expression scores in representative BCCL (a composite of data from the Sanger, GSK, and Neve et al. datasets, see Methods) and a human breast tumor dataset. *** p < 1E-10 (F-test for difference in variance in module score). (A,C,E) These box plots show the distributions of Pearson correlation coefficients for all pairs of genes in each module, respectively, for the BCCL and tumor datasets. ***Modules 4-Immune, 5-Immune, and 9-ECM/Dev/Immune can be considered tumor-extrinsic, as their constituent genes are uncorrelated in BCCLs but highly correlated in patient tumor biopsies in all datasets tested (median r>0.35). Datasets tested: GSE21653 (Figure 4), GSE1456, GSE2034, GSE3494.

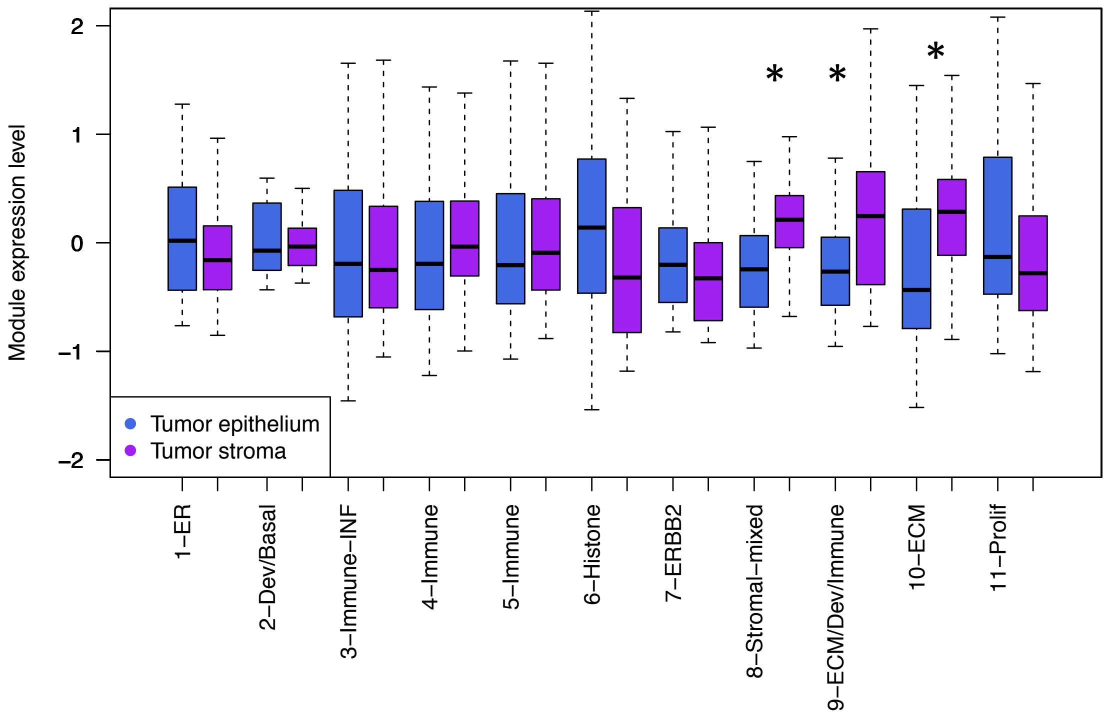

Figure S5: Module expression in microdissected tumor stroma vs. epithelium. We used the dataset GSE5847 to compare module expression levels in micro-dissected tumor epithelium and stroma. Only ECM/stromal modules 8-10 had significantly different expression levels (BH p-value <0.05).

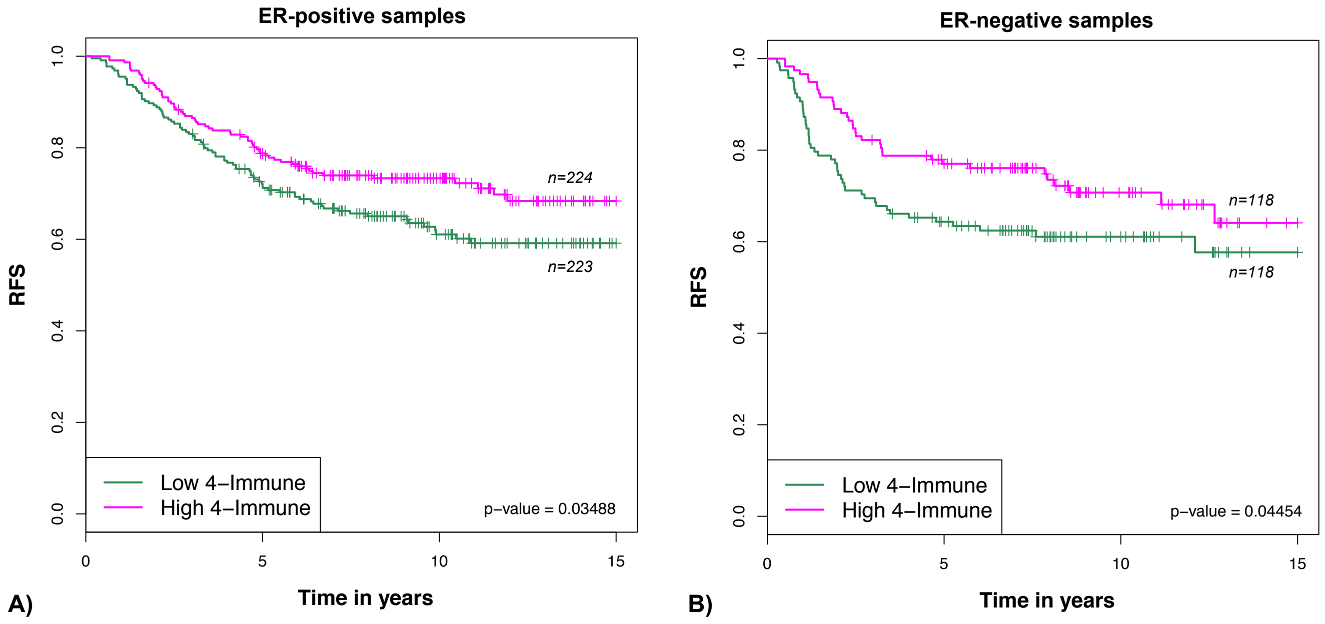

Figure S6: Upregulation of a T cell/B cell immune module was associated with RFS in ER+ and ER- subsets. These Kaplan-Meier plots show that T cell/B cell immune module 5-immune is significantly associated with RFS in ER+ and ER- patient subsets in our pooled dataset of 683 node-negative adjuvantly untreated cases. Module expression was dichotomized at the median.

	Univariate Cox RFS analysis		Multivariate Cox - adjusted for ER and Her2		Multivariate Cox - adjusted for ER, Her2 & 11-Prolif	
	HR (95% CI)	BH p-val	HR (95% CI)	BH p-val	HR (95% CI)	BH p-val
**1-ER**	0.6299 (0.4730, 0.8389)	**0.0076**	0.69381 (0.5206, 0.9247)	**0.0367**	0.9886 (0.7428, 1.3156)	0.95769
**2-Dev/Basal**	1.1119 (0.7019, 1.7613)	0.746	0.9988 (0.6300, 1.5836)	0.996	0.9121 (0.5856, 1.4205)	0.8535
**3-Immune-INF**	1.0134 (0.8767, 1.1714)	0.8572	0.9972 (0.8618, 1.1538)	0.996	0.8832 (0.7634, 1.0219)	0.1854
**4-Immune**	0.7482 (0.6134, 0.9127)	**0.0088**	0.7224 (0.5917, 0.8820)	**0.0054**	0.6818 (0.5571, 0.8344)	**0.00096**
**5-Immune**	0.8299 (0.6866, 1.0031)	0.1103	0.7976 (0.6596, 0.9646)	**0.0385**	0.7012 (0.5789, 0.8495)	**0.00096**
**6-Histone**	1.1445 (0.9912, 1.3214)	0.123	1.141 (0.9874, 1.3185)	0.1376	1.1012 (0.9494, 1.2773)	0.3401
**7-ERBB2**	1.0733 (0.9558, 1.2052)	0.3801	1.0268 (0.9148, 1.152)	0.8069	1.0343 (0.9241, 1.1577)	0.80159
**8-Stromal-mixed**	1.0682 (0.7823, 1.4586)	0.746	1.0727 (0.7845, 1.466)	0.8069	1.3262 (0.9713, 1.8107)	0.1854
**9-ECM/Dev/Immune**	0.6642 (0.5077, 0.8688)	**0.0076**	0.6825 (0.5212, 0.8936)	**0.0155**	1.0072 (0.7720, 1.3142)	0.95769
**10-ECM**	1.0658 (0.8906, 1.2756)	0.6709	1.0878 (0.9078, 1.3036)	0.5713	1.3393 (1.1187, 1.6033)	**0.0059**
**11-Prolif**	1.6721 (1.3856,2.0179)	**1.10E-06**	1.5936 (1.32, 1.9238)	**1.12E-05**	-	-

Table S2: Recurrence free survival analysis of the pooled prognostic dataset of 683 node-negative adjuvant untreated cases (see Methods). We used Cox survival models, both with and without adjustment for ER and Her2 status and proliferation (11-Prolif expression), to assess associations between module expression and RFS in this population.

Logistic model of pCR: GSE22093					
Module	Coeff:module	Ward p-value	BH Ward p-value	ANOVA P(>|Chi|)	AUC of lgr
1_ER	-1.669	**0.00413**	**0.01514**	**0.00168**	0.71264
2_Dev/Basal	1.081	0.23302	0.32040	0.23235	0.61422
3_Immune-INF	1.023	**0.00308**	**0.01514**	**0.00128**	0.7342
4_Immune	0.815	**0.01401**	0.06787	**0.00924**	0.65158
5_Immune	1.072	**0.00359**	**0.01724**	**0.00226**	0.68966
6_Histone	-0.104	0.7101	0.71010	0.70752	0.49282
7_ERBB2	-0.221	0.4255	0.46348	0.41327	0.5819
8_Stromal-mixed	1.222	**0.03702**	**0.03082**	**0.02811**	0.66523
9_ECM/Dev/Immune	0.369	0.37921	0.46805	0.38255	0.57399
10_ECM	0.400	0.22327	0.32040	0.22088	0.62356
11_Prolif	1.056	**0.00627**	**0.01514**	**0.0039**	0.70115

Table S3: Associations between module expression and pCR. We used a logistic regression modeling and ROC analysis to assess associations between module expression and chemotherapy response of patients from GSE22093 who received neoadjuvant chemotherapy. BH: Benjamini-Hochberb multiple testing correction applied. AUC: area under the curve, a measure of predictive accuracy. ANOVA: analysis of variance.

Logistic model pCR~ moduleA + moduleB: GSE22093					
Module pair (moduleA-moduleB)	Coeff:moduleA	Ward p-value	Coeff:moduleB	Ward p-value	AUC of lgr
4_Immune-11_Prolif	1.0155	**0.00641**	1.28521	**0.00299**	0.7967
5_Immune-11_Prolif	1.07637	**0.00642**	1.04804	**0.01123**	0.77371
3_Immune-10_ECM	1.09359	**0.00234**	0.56552	0.12886	0.76652
1_ER-3_Immune	-1.27394	**0.03588**	0.8301	**0.02639**	0.76006
8_Mixed-11_Prolif	1.48902	**0.02771**	1.14757	**0.00466**	0.75934
9_ECM/Dev/Immune-11_Prolif	1.1447	**0.02578**	1.48574	**0.00134**	0.75575
3_Immune-8_Mixed	0.94965	**0.00768**	0.95986	0.11742	0.75144
10_ECM-11_Prolif	0.74184	0.05592	1.28675	**0.00249**	0.75072
3_Immune-11_Prolif	0.91142	**0.01199**	0.9059	**0.02971**	0.74928
3_Immune-4_Immune	0.90281	**0.01271**	0.58281	0.07811	0.74713
3_Immune-6_Histone	1.08802	**0.00239**	-0.29307	0.35048	0.74497
3_Immune-5_Immune	0.75051	0.05336	0.68226	0.09786	0.74066
1_ER-9_ECM/Dev/Immune	-1.71505	**0.00437**	0.40458	0.36333	0.73922
3_Immune-9_ECM/Dev/Immune	1.06039	**0.0027**	0.47903	0.27256	0.73922
2_Dev/Basal-3_Immune	0.82096	0.39139	1.0077	**0.00406**	0.73779
1_ER-4_Immune	-1.36734	**0.03035**	0.48663	0.16924	0.73491
3_Immune-7_ERBB2	1.02376	**0.00388**	0.00193	0.99445	0.7342
1_ER-5_Immune	-1.12613	0.10801	0.64063	0.15133	0.73204
1_ER-11_Prolif	-1.18015	0.09121	0.56634	0.2292	0.72989
1_ER-2_Dev/Basal	-2.11372	**0.00379**	-1.27674	0.30443	0.71839

Table S4: Associations between module pairs and pCR. We used a multivariate logistic regression modeling and ROC analysis to assess associations between expression of pairs of modules and chemotherapy response of patients from GSE22093 who received neoadjuvant chemotherapy. BH: Benjamini-Hochberb multiple testing correction applied. AUC: area under the curve, a measure of predictive accuracy. lgr: logistic regression model.

Site of metastasis analysis (N=157 met to bone + 67 met to lung or brain = 224 primary tumors)							
	Univariate analysis				Multivariate: adjusted for ER and ERBB2		
**Module**	**Association**	**BH p-value**	**AUC**	**Mean diff (Bone - Lung/brain)**	**p-value**	**BH p-value**	**AUC**
**1-ER**	Bone	**3.05E-11**	**0.8113**	**0.5445**	**0.0069**	0.0759	**0.813**
**2-Dev/Basal**	Lung/brain	**2.65E-08**	**0.7479**	**-0.2853**	0.3239	0.4803	0.7962
**3-Immune-INF**	Lung/brain	**0.01165**	**0.6178**	**-0.3229**	0.0891	0.2367	0.8053
**4-Immune**	Lung/brain	**0.01165**	**0.6154**	**-0.2998**	0.393	0.4803	0.7955
**5-Immune**	Lung/brain	**0.000154**	**0.6646**	**-0.4328**	0.1076	0.2367	0.7994
**6-Histone**	-	0.08496	0.579	0.2702	0.2711	0.4803	0.7986
**7-ERBB2**	-	0.08496	0.6501	0.27	0.383	0.4803	0.7964
**8-Stromal-mixed**	Lung/brain	**0.002583**	**0.6294**	**-0.2225**	0.8314	0.8314	0.7955
**9-ECM/Dev/Immune**	Bone	**0.01165**	**0.6318**	**0.2238**	**0.0201**	0.1106	**0.817**
**10-ECM**	-	0.09192	0.5736	0.1703	0.6908	0.7599	0.7972
**11-Prolif**	Lung/brain	**1.42E-06**	**0.7352**	**-0.5698**	**0.03554**	0.1303	**0.8121**

Table S5: Site of metastasis analysis. This table summarizes associations between module expression and site of metastasis among women in our pooled dataset who suffered distant recurrence. We used logistic regression modeling, with and without adjustment for ER and ERBB2 expression, to assess associations. This analysis used a pooled dataset of 572 samples from 3 GEO data sets (GSE2034, GSE2603, GSE12276), pre-processed as described in Methods.

	Bone-specific RFS analysis					
	RFS analysis using patients who recurred in bone-only (N=157) and those without recurrence (N=311)				Time to bone-specific recurrence (N=157 pts who developed bone mets)	
	Univariate analysis		Multivariate: adjusted for ER and ERBB2		Univariate analysis	
**Module**	**Bone-specific RFS association**	**BH p-value**	**p-value**	**BH p-value**	**Association**	**BH p-value**
**1-ER**	Decreased Bone-RFS	**0.011143**	0.8959	0.9247	Longer time to recurrence	**0.0008213**
**2-Dev/Basal**	Increased Bone-RFS	**0.001565**	0.1866	0.3357	-	0.3033
**3-Immune-INF**	-	0.06277	0.1889	0.3357	-	0.3497
**4-Immune**	Increased Bone-RFS	**0.008483**	0.1139	0.3294	-	0.9300
**5-Immune**	Increased Bone-RFS	**0.005303**	0.117	0.3294	-	0.7637
**6-Histone**	-	0.7199	0.9246	0.9247	-	0.9300
**7-ERBB2**	-	0.7625	0.8992	0.9247	-	0.2021
**8-Stromal-mixed**	-	0.9076	0.12	0.3294	-	0.9879
**9-ECM/Dev/Immune**	-	0.7625	0.7816	0.9247	-	0.4773
**10-ECM**	Decreased Bone-RFS	**0.008483**	**0.004339**	**0.04421**	-	0.7637
**11-Prolif**	-	0.7624	0.2265	0.3611	Shorter time to recurrence	**0.0008213**

	Lung-specific RFS analysis					
	RFS analysis using patients who recurred in lung-only (N=44) and those without recurrence (N=311)				Time to lung-specific recurrence (N=44 pts who developed lung mets)	
	Univariate analysis		Multivariate: adjusted for ER and ERBB2		Univariate analysis	
**Module**	**Lung-specific RFS association**	**BH p-value**	**p-value**	**BH p-value**	**Association**	**BH p-value**
**1-ER**	Increased Lung-RFS	**1.83E-06**	0.2732	0.5229	Longer time to recurrence	**0.009482**
**2-Dev/Basal**	Decreased Lung-RFS	**0.003428**	0.8243	0.824	Shorter time to recurrence	**0.01428**
**3-Immune-INF**	-	0.7913	0.5285	0.6414	-	0.4774
**4-Immune**	Increased Lung-RFS	0.2218	**0.01578**	0.09068	-	0.8303
**5-Immune**	-	0.9561	0.05218	0.1519	-	0.9101
**6-Histone**	-	0.0757	0.2887	0.5229	-	0.7410
**7-ERBB2**	-	0.2218	0.6584	0.7327	-	0.7410
**8-Stromal-mixed**	Decreased lung-RFS	**0.00436**	0.3338	0.53	-	0.6254
**9-ECM/Dev/Immune**	Increased lung-RFS	**0.003428**	**0.03963**	0.14	-	0.6254
**10-ECM**	-	0.8199	0.521	0.6414	-	0.1014
**11-Prolif**	Decreased lung-RFS	**4.28E-05**	0.1125	0.302	Shorter time to recurrence	**0.009482**

	Brain RFS analysis					
	RFS analysis using patients who recurred in brain (N=31) and those without recurrence (N=311)			Time to brain recurrence (N=31 pts who developed brain mets)		
	Univariate analysis		Multivariate: adjusted for ER and ERBB2	Univariate analysis		
**Module**	**Brain RFS association**	**BH p-value**	**p-value**	**Association**	**p-value**	**BH p-value**
**1-ER**	Increased Brain-RFS	**0.003758**	0.1566	Longer time to recurrence	**0.03144**	0.1357
**2-Dev/Basal**	Decreased Brain-RFS	**0.032854**	0.1869	Shorter time to recurrence	**0.01691**	0.1357
**3-Immune-INF**	-	0.8262	0.2594	-	0.4870	0.7041
**4-Immune**	-	0.1005	0.2903	-	0.9802	0.9802
**5-Immune**	-	0.0982	0.3246	-	0.6186	0.7560
**6-Histone**	-	0.4618	0.6316	-	0.4766	0.7041
**7-ERBB2**	-	0.5069	0.6664	Shorter time to recurrence	**0.03701**	0.1357
**8-Stromal-mixed**	Decreased Brain-RFS	**0.02717**	0.7696	-	0.5121	0.7041
**9-ECM/Dev/Immune**	-	0.6522	0.8807	-	0.8412	0.9254
**10-ECM**	-	0.4389	0.9309	-	0.5085	0.7041
**11-Prolif**	Decreased Brain-RFS	**0.02717**	0.9923	-	0.1037	0.2850

Table S6: Site-specific RFS analysis. This table summarizes associations between module expression and site-specific RFS, using Cox survival analysis with and without adjustment for ER and ERBB2 expression. Also included is time to recurrence analysis for the women who recurred to each organ site. This analysis used a pooled dataset of 572 samples from 3 GEO data sets (GSE2034, GSE2603, GSE12276), pre-processed as described in Methods.
